# Advances and Clinical Translation Potentials of Functional Nanomaterials in Tissue Engineering

**DOI:** 10.3390/bioengineering13080902

**Published:** 2026-08-10

**Authors:** Yuhan He, Qiang Peng

**Affiliations:** State Key Laboratory of Oral Diseases & National Center for Stomatology & National Clinical Research Center for Oral Diseases, West China Hospital of Stomatology, Sichuan University, Chengdu 610041, China

**Keywords:** tissue repair, regenerative medicine, drug delivery, bioengineering, artificial intelligence

## Abstract

Functional nanomaterials, such as functionalized nanoparticles, nanofibers, nanocrystals, MXene and liposomes, have emerged as game-changers in tissue engineering, enabling precise modulation of cellular behaviors and dynamic biomimetic microenvironments. This review comprehensively summarizes and discusses the cutting-edge applications of nanomaterials in tissue regeneration (including bone, skin, neural and cardiac tissue regeneration), with a focus on their unique physicochemical properties (e.g., stimuli-responsiveness, nano-topography) and hybrid system design. Recent breakthroughs include 4D-printed shape-memory nanocomposites for irregular bone defects and “smart” wound dressings integrating antibacterial nanoparticles with real-time biosensing. However, clinical adoption remains constrained by unresolved challenges in biocompatibility, scalability of nanomanufacturing, and regulatory ambiguities. We critically analyze these barriers and propose a translational roadmap leveraging AI-driven material design and multi-omics validation platforms to accelerate commercialization.

## 1. Introduction

As an interdisciplinary frontier integrating material science, biology, engineering, clinical medicine and other fields, tissue engineering has become a central component of regenerative medicine [1]. Over the past decades, it has been used to treat tissue defects caused by trauma, diseases, and congenital factors. It has made significant progress and is now a thriving research area. Tissue engineering primarily utilizes cells, scaffolds and bioactive factors to construct biological substitutes in vivo or in vitro, aiming to restore, replace, or regenerate damaged tissues and organs [2,3,4]. Conventional approaches to tissue repair, including autologous and allogeneic transplantation, remain limited by donor scarcity, strong immune rejection, low compatibility rates, and variable clinical outcomes [5,6]. These constraints continue to drive the search for more effective regenerative strategies.

An avenue for innovation of tissue engineering resides in the soaring development of nanomaterials. Nanomaterials are materials with microstructural dimensions in the range of 1 to 100 nanometers and functional nanomaterials have become the ideal candidates for tissue engineering [7]. At this scale, materials often exhibit physicochemical characteristics that differ markedly from those of their bulk counterparts, namely the same materials in their conventional macroscale or non-nanoscale form. Compared with these bulk forms, nanomaterials can display distinct surface effects, tunable mechanical behavior, and favorable biointerface properties [8,9]. These features make functional nanomaterials well suited for regulating cellular behavior, constructing biomimetic microenvironments, exerting antibacterial and anti-inflammatory effects, and enabling targeted drug delivery [10,11,12,13,14]. As a result, functional nanomaterials have emerged as pivotal tools in modulating the complex pathological microenvironment and better repairing tissue damage such as bone, skin and neural injuries [15,16,17].

**Figure 1:**
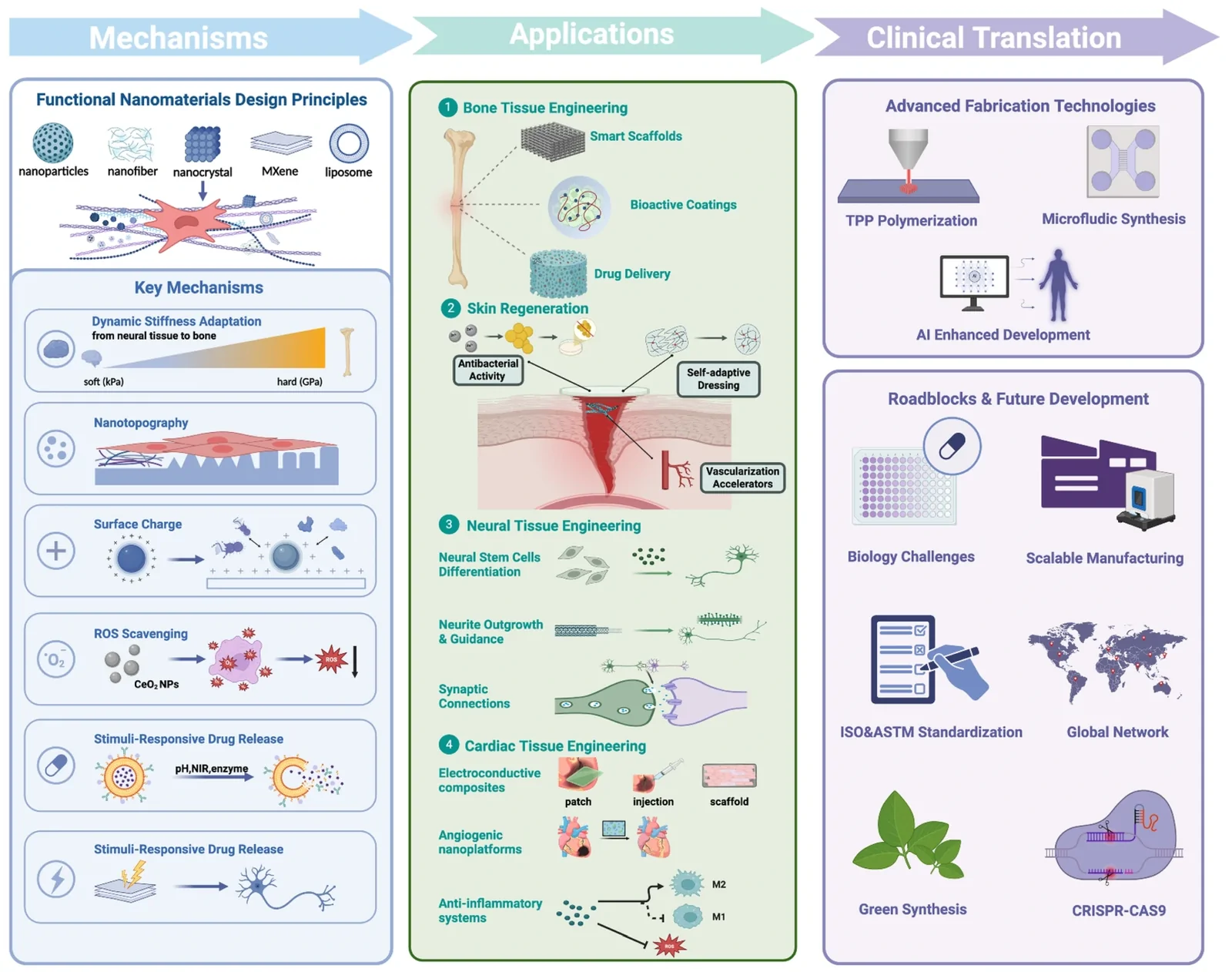
Overview of functional nanomaterials in tissue engineering. The figure summarizes the major design principles and biological mechanisms of functional nanomaterials, including nanoparticles, nanofibers, nanocrystals, MXene, and liposomes, as well as dynamic stiffness adaptation, nanotopographical regulation, surface charge-mediated interactions, ROS scavenging, and stimuli-responsive drug release. Representative applications in bone, skin, neural, and cardiac tissue engineering are illustrated, together with advanced fabrication technologies and the key challenges for clinical translation.

This review emphasizes a translationally oriented framework for functional nanomaterials in tissue engineering (Figure 1). We summarize recent advances from the perspective of structure–function relationships, key regenerative properties, and multifunctional system design, thereby linking nanoscale features with biological performance. In addition, this review integrates emerging fabrication strategies, including two-photon polymerization, microfluidic synthesis, and AI-assisted design, with applications in bone, skin, neural, and cardiac regeneration. Beyond outlining progress in therapeutic advances, we place particular emphasis on the barriers that prevent many nanomaterial systems from moving beyond preclinical studies. By unifying material design, fabrication, regenerative application, and translational feasibility within one framework, this review aims to provide a more clinically relevant perspective for the future development of functional nanomaterials.

## 2. Nanomaterial Design Paradigms in Tissue Engineering

In order to understand the expanding roles of nanomaterials in tissue engineering, it is necessary to consider the principles that guide their design. In this context, rational design is not simply a matter of material selection, but of establishing a clear relationship between structural characteristics and regenerative function. This section outlines the main design considerations that underlie the use of functional nanomaterials in tissue repair, with emphasis on structure–function relationships and the key properties that support tissue regeneration.

### 2.1. Structure–Function Relationships

A central issue in the design of nanomaterials for tissue engineering is the relationship between structure and function. Subtle differences in material structure can lead to marked changes in cell adhesion, migration, differentiation, and tissue response. Parameters such as size, morphology, stiffness, and surface characteristics jointly determine their biological performance in tissue engineering.

#### 2.1.1. Nanotopography-Guided Cell Fate

**Figure 2:**
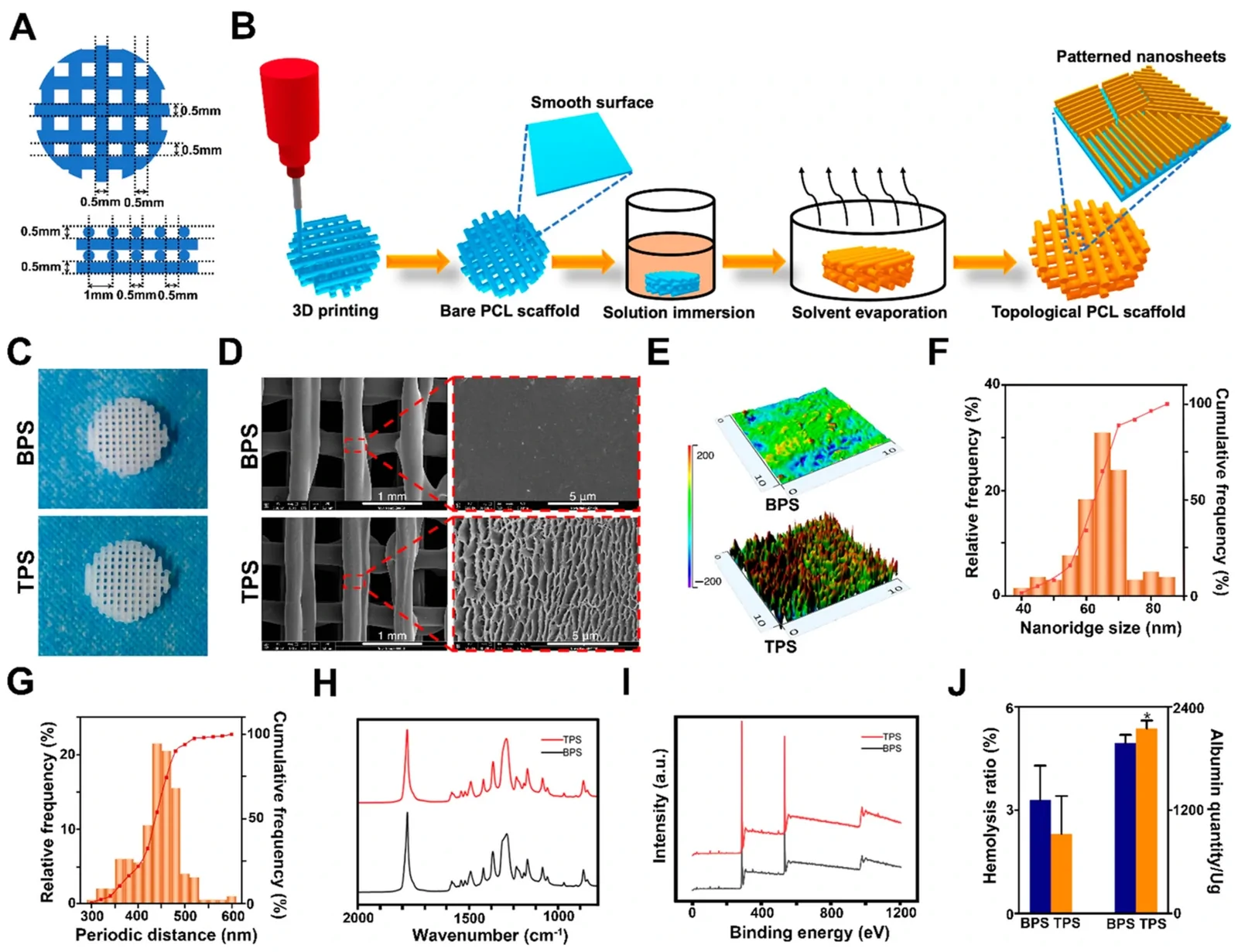
A representative example of nanotopography-engineered 3D-printed PCL scaffolds used to provide biomimetic physical cues for cell-material interactions. (**A**) Structural design of the scaffold. (**B**) Fabrication strategy for generating surface nanotopography on 3D-printed PCL scaffolds. (**C**–**E**) Gross morphology and micro/nanoscale surface features of bare and topologically modified scaffolds, as characterized by photographic imaging, SEM, and AFM. (**F**,**G**) Quantitative analysis of nanoridge dimensions and periodic spacing on the modified surface. (**H**,**I**) FTIR and XPS characterization showing that the topological treatment primarily alters surface architecture rather than the bulk chemical composition. (**J**) Comparison of hemolysis ratio and albumin adsorption between the two scaffold types. * *p* < 0.05. Reprinted with permission from Ref. [21].

Among structural features, morphology and surface nanotopography are particularly important as they directly shape how cells perceive and respond to the surrounding microenvironment [8,18,19,20]. Nanoscale features such as roughness, grooves, aligned fibers, and pores can provide physical cues resembling the native extracellular matrix (ECM). Representative examples include topologically engineered 3D-printed PCL scaffolds, in which nanosheet-based surface modulation generates architectures without altering the underlying chemical composition, thereby offering defined physical cues for cell-material interactions (Figure 2) [21]. In neural tissue engineering, for example, conductive nanocomposite hydrogels incorporating different dimensions of nanomaterials have nanoscale topographical features such as surface roughness and aligned fibrillar patterns. These features can simulate the complex structure of neural ECM and enhance cellular responses to propel neural regeneration [22].

Similar effects have been reported in orthopedic applications. Titanium-based implants are widely used because of their favorable biocompatibility. TiO_2_ nanotubes and nanogroove structures on implant surfaces not only exhibit excellent bioactivity and adjustability, but also modulate cell adhesion and response, upregulate the expression of angiogenesis-related genes (e.g., GLI1) and growth factors (e.g., VEGF) [23,24,25]. Nanotopographical cues are also closely associated with stem cell fate regulation. Xing et al. reported that nanotopographical 3D-printed polycaprolactone (PCL) scaffolds promoted the proliferation and osteogenic gene expression of urine-derived stem cells (USCs) and provided a microenvironment resembling native bone tissue [19,21].

In another example, a single-step spin coating approach was used to create nanotopography on 3D-printed porous polylactic acid (PLA) scaffolds. The nanoscale topography endows them with antibacterial abilities and osteogenic activities [24]. Compared with unmodified scaffolds, the nanostructured PLA surface showed strong contact-killing activity against P. aeruginosa (86.60% dead cells in 24 h) and *S. aureus* (92.36%), while also supporting the adhesion and proliferation of pre-osteoblasts and promoting osteogenic differentiation more effectively [26]. Taken together, these findings suggest that nanotopography is not merely a structural feature, but a design element that can be used to direct cellular behavior in a predictable manner.

#### 2.1.2. Surface-Property-Mediated Biological Outcomes

Beyond the well-recognized roles of nanotopography and morphology, surface properties serve as the equally important tools bridging nanoscale structure to their functions. Nanomaterials possess a high surface-to-volume ratio, which renders surface properties as the primary regulator of nanomaterials’ performance [27]. Surface chemistry, charge, wettability, and ligand presentation can all influence protein adsorption, cellular uptake, immune recognition, and therapeutic efficacy.

For instance, surface functionalization is often used to improve targeting and drug delivery performance. A Toll-like receptor 9 agonist, CpG, has been adsorbed onto the surface of ZANPs to enhance immune response while delivering antigens [28]. Surface charge can contribute directly to antibacterial activity. A zinc-doped hydroxyapatite/chitosan-gelatin nanocomposite scaffold carries positive charges derived from chitosan and zinc ions, which interact with negative charges of bacterial peptidoglycan. This interaction disrupts bacterial cell membrane and significantly improves the antibacterial activity against *E. coli* (inhibition zone: 11 ± 0.5 mm) and *S. aureus* (inhibition zone: 13 ± 0.45 mm) [29].

Furthermore, surface properties also govern the cellular uptake pathways. Wu et al. found that graphene oxide (GO) exhibits strong binding affinity with low-density lipoprotein (LDL), and macrophages prefer taking up more GOs in the presence of LDL [30]. This observation highlights how the surface characteristics of nanomaterials may alter their interactions with biomolecules and thereby influence cellular uptake and downstream biological responses.

Overall, the structure–function relationship reveals the cause and effect between physicochemical properties and biological effects in nanomaterial design. Although various nanomaterials may act through distinct mechanisms, their biological performance ultimately depends on how physicochemical features are translated into cellular and tissue-level effects. A clear understanding of this relationship is essential for the rational development of regenerative nanomaterials.

### 2.2. Key Properties Driving Regeneration

Beyond structural design, several functional properties are significant for successful tissue regeneration. In practice, nanomaterials are rarely designed for a single isolated feature. Instead, they are expected to match the mechanical, biochemical, and in some cases electrical requirements of the target tissue. Among these considerations, dynamic mechanical adaptability, signaling modulation, and multifunctional integration are particularly relevant.

#### 2.2.1. Dynamic Mechanical Adaptability

It’s acknowledged that dynamic mechanical adaptability plays a vital role in nanomaterials’ design. Native tissues span a broad range of stiffness, from less than 1 kPa in brain tissue to more than 1 GPa in bone. Cells can respond to stimuli such as the stiffness, elasticity, and nanotopography of the extracellular matrix (ECM), then convert them into biological signals to regulate various cellular behaviors [31]. Therefore, nanomaterials used in tissue engineering should be designed to match the mechanical environment of the target tissue as closely as possible.

For neural applications, a β-peptide named betaVhex has been assembled with carbon nanotubes (CNTs) to form a soft conducive hydrogel with excellent biocompatibility and stability. Its Young’s modulus is close to that of brain tissue (approximately 1 kPa), so it can be tightly connected to neural tissue and significantly reduces the mechanical mismatch at the neural interface [32]. In skin repair, a GelMA/HA-E/Ag@MOF composite hydrogel loaded with silver nanoparticles through cyclodextrin metal–organic frameworks exhibited a modulus of 125 kPa with 1% HA-E addition, consistent with the mechanical demands of skin. In addition, the sustained release of silver ions endowed the material with both antibacterial and anti-inflammatory functions, contributing to improved burn wound healing [33]. For bone regeneration, a 3D-printed PCL/nHA composite scaffold reached optimal stiffness at 30% nHA content, with a tensile modulus of 506.6 MPa and a compressive modulus of 56.43 MPa, which are close to the mechanical properties of native bones. This scaffold was shown to regulate cell behavior, reduce inflammation, and promote bone regeneration, representing a promising implant for in situ bone regeneration [34].

These examples illustrate that mechanical adaptation is more than a structural support. Properly tuned nanomaterials can actively influence the cellular microenvironment and help create conditions for tissue regeneration.

#### 2.2.2. Signaling Modulation

Various indicators at the site of tissue injury, such as ROS levels, inflammatory factor levels, macrophage polarization, and vascular regeneration, all affect tissue repair. One major advantage of functional nanomaterials is their ability to modulate these signals in a controlled manner, thereby improving the pathological microenvironment at the injury site.

Cerium oxide nanoparticles are a representative example. Because they can mimic the activities of antioxidant enzymes such as superoxide dismutase (SOD) and catalase (CAT), they are able to reduce excessive reactive oxygen species (ROS) and alleviate oxidative damage. This ability of cerium oxide nanoparticles to maintain redox homeostasis in pathological conditions enables them to regulate oxidative stress during regeneration, showing great potential in tissue engineering. However, it should also be noted that the biological effects of CeO_2_ nanoparticles are dose-dependent, and high concentrations may induce cytotoxicity, including liver injury, cell damage, and apoptosis [35].

Zinc oxide nanoparticles synthesized by either chemical or physical methods also participate in signaling regulation through multiple mechanisms. Under UV irradiation, they generate low concentrations of ROS such as hydroxyl radicals and hydrogen peroxide, which activate the MAPK/AKT signaling pathway to promote angiogenesis and accelerate wound healing. At the same time, they can modulate the secretion of inflammatory factors like TNF-α and IL-6 and anti-inflammatory factors like IL-4. Meanwhile, they can also promote macrophage polarization toward the anti-inflammatory M2 phenotype, thereby helping to suppress excessive inflammation [36]. Another example is tFNA-Que, which can reduce pathogen lipopolysaccharide (LPS)-induced ROS production through the Nrf2/HO-1 pathway and to regulate macrophage polarization by affecting NF-κB nuclear translocation. Hence, this material exerts both antioxidant and anti-inflammatory effects and promotes wound healing under inflammatory conditions [37,38].

#### 2.2.3. Multifunctional Integration

Tissue repair typically involves multiple biological processes, and there is increasing interest in nanomaterials capable of integrating several functions within a single platform. Such platforms can simultaneously provide structural support, conductivity, responsiveness to external stimuli, and regulation of cell behavior, making it possible to efficiently induce tissue reconstruction. This trend toward multifunctional design has become prominent in the engineering of complex or electroactive tissues.

**Figure 3:**
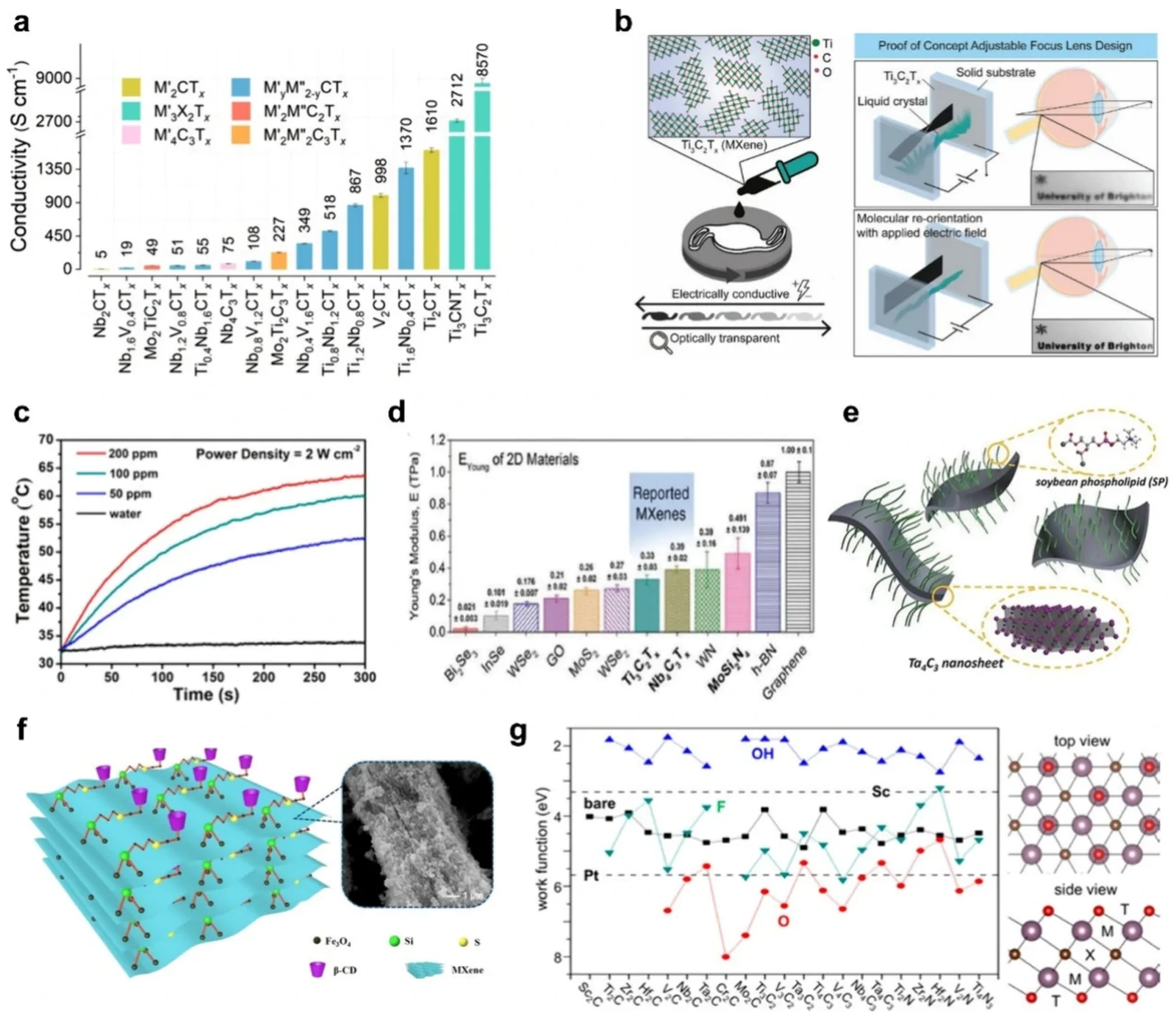
Representative physicochemical properties of MXenes relevant to biomedical applications. (**a**) Electrical conductivity of different MXene compositions. (**b**) Conceptual design of an adjustable-focus lens based on the electrical conductivity and optical transparency of Ti_3_C_2_T_x_ MXene. (**c**) Photothermal heating profiles of MXene dispersions at different concentrations under laser irradiation. (**d**) Young’s modulus of representative 2D materials, highlighting the mechanical properties of MXenes. (**e**,**f**) Surface area, layered structure, and surface functionalization of MXenes for drug loading and delivery. (**g**) Tunable chemical properties of MXenes through surface termination and modification. Reprinted with permission from Ref. [17].

MXene-based composites are among the most widely studied multifunctional nanomaterial systems (Figure 3). Owing to their favorable electrical conductivity, mechanical properties, and tunable surface chemistry, they can satisfy the demands of electroactive tissues to promote repair and regeneration [39,40]. Electrospun MXene-PCL composite scaffolds exhibit good conductivity, mechanical properties and biocompatibility. They can mimic the electrical microenvironment of native tissues and provide suitable electrical stimulation for cells. As a result, they support cell proliferation and differentiation, and are considered promising for neural and cardiac regeneration [41]. In cardiac tissue engineering, 3D-printed MXene-PEG composite cardiac patches have been developed to mimic both the structure of the native ECM and the conductivity of the myocardial tissue. These patches provide both conductive and topographical cues for human induced pluripotent stem cell derived cardiomyocytes (iCMs), enabling the treatment of myocardial infraction [42]. In the nervous system, MXene-Matrigel hydrogels combine high conductivity with a 3D porous network. They offer an ECM-like matrix that supports neural stem cell growth and promotes neuronal differentiation [43].

Multifunctional integrated systems exemplified by MXene-based composites are valuable for tackling the complex demands of tissue repair. Instead of relying on a single property, these composite nanosystems are designed to coordinate regenerative cues within the same platform, which may improve their overall therapeutic performance. Despite their attractive conductivity, antibacterial activity, and photothermal responsiveness, MXene-based materials remain at a relatively early translational stage. Their in vivo degradation behavior is not yet standardized, and available biosafety conclusions are strongly influenced by synthesis route, oxidation state, flake size, dose, and surface chemistry. Therefore, although MXenes are promising multifunctional platforms, their translational readiness is currently less mature than that of more established localized scaffolds or coating systems.

## 3. Applications in Regenerative Medicine

### 3.1. Bone Tissue Engineering

Bone is the most important supporting organ in the human body and possesses a certain capacity for self-repair owing to its composite organic and inorganic architecture. However, bone defects caused by trauma, infection, tumor resection, and congenital factors remain a major challenge. Such defects not only affect patients’ quality of life, but may also lead to permanent functional impairment [44]. Autologous bone transplantation is the gold standard for treating bone defects, but its clinical use is limited by restricted donor availability and donor-site morbidity [45]. Although allografts can overcome the donor shortage, they are associated with the risk of immune rejection, and the variability of osteogenic induction does exist [46]. Consequently, there is an urgent demand for effective bone substitutes. Nanomaterials have emerged as a promising direction for bone repair because of their favorable bioactivity, structural biomimicry, dynamic responsiveness, and suitability for personalized bone defects [47].

#### 3.1.1. Smart Scaffolds

To meet the 3D shape requirements for bone defect repair, nanomaterials have been designed as bone substitutes. Traditional 3D-printed bone scaffolds have become increasingly mature, demonstrating multiple properties such as good mechanical performance, osteogenic activity, and biocompatibility [48]. However, most of these scaffolds respond passively to the surrounding environment, failing to match dynamic physiological processes after implantation. In contrast, 4D-printed shape-memory smart scaffolds, which introduce a temporal dimension based on 3D printing, demonstrate both adaptability and stimuli responsiveness.

4D-printed MXene composite scaffolds can dynamically respond to external stimuli such as light, heat, magnetism, and electricity, showing great potential in bone and cartilage repair [49]. For example, 4D-printed PLA-PBAT-Fe_3_O_4_ nanocomposites can be driven to a desired shape by an external magnetic field owing to their magnetic response properties. And they are thus fabricated for personalized bone defect treatment. In addition, 4D printing can fabricate smart scaffolds with complex surface nanopatterns, which possess the functions of inducing osteogenic differentiation, antibacterial activity and immunoregulation [50]. These features suggest that 4D printed structures may offer a more robust solution for tissue growth. Despite all the merits, 4D printing technology is still under development. Further research is required to clarify how 4D-printing materials respond to external stimuli, how they interact with cells, and how 4D printed scaffolds achieve reversible transformation [51].

#### 3.1.2. Bioactive Coatings

**Figure 4:**
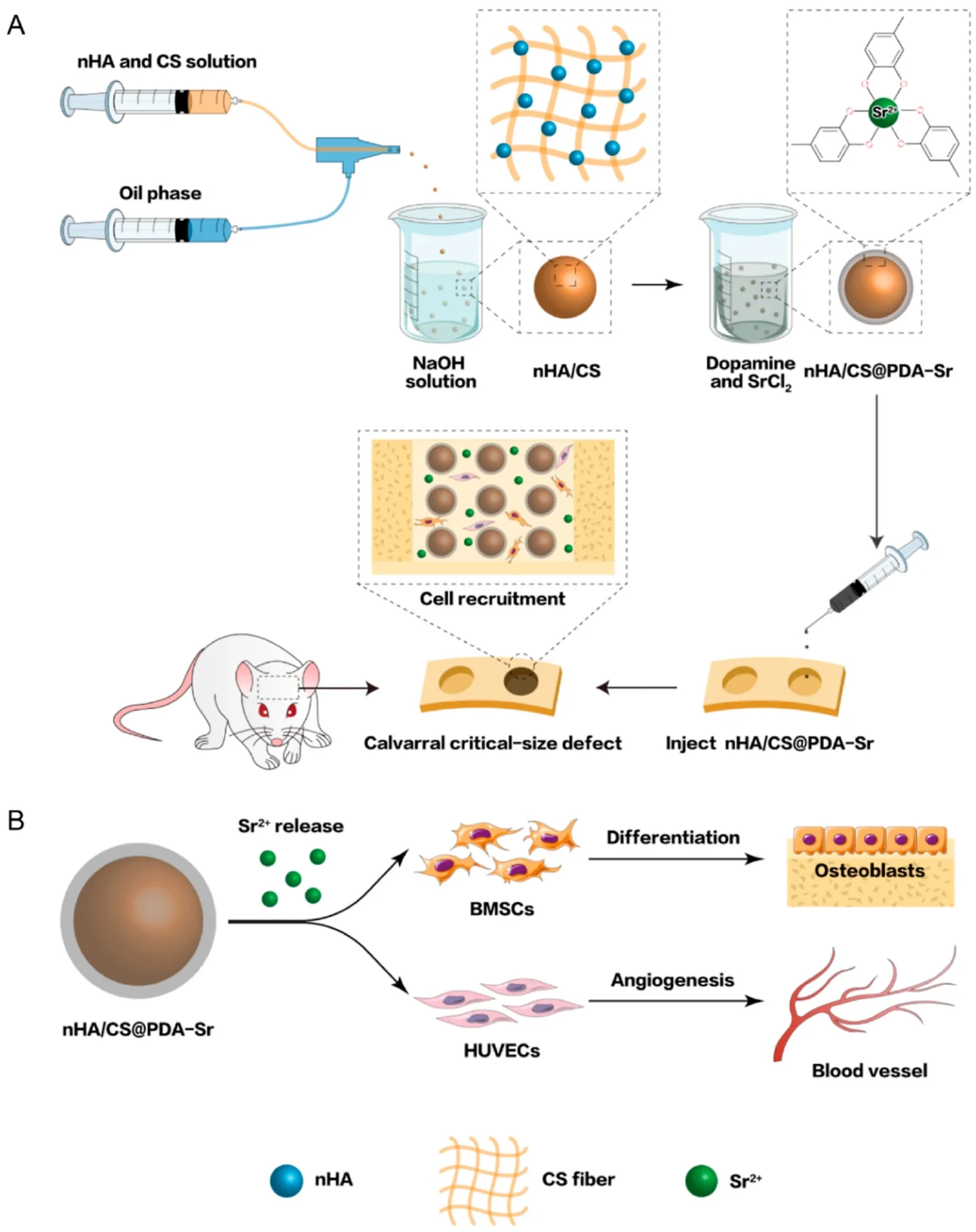
Schematic diagram of the fabrication and bone regeneration mechanism of strontium ion-functionalized nano-hydroxyapatite/chitosan composite microspheres (nHA/CS@PDA-Sr). (**A**) nHA/CS@PDA-Sr microspheres are prepared by constructing nHA/CS microspheres and subsequent Sr-functionalization, and then injected into a critical-size calvarial defect. (**B**) The microspheres recruit cells and release Sr^2+^, which promotes BMSC osteogenic differentiation and HUVEC angiogenesis, thereby accelerating vascularized bone repair. Reprinted with permission from Ref. [55].

In addition to smart scaffolds, bioactive coatings represent another effective approach for promoting osteogenesis. Hydroxyapatite (HA), the main mineral component of natural bone, is widely used in bone graft materials because nano-hydroxyapatite can improve material bioactivity [52,53]. Strontium is an essential trace element for bone. It can regulate osteogenic differentiation and stimulate osteoblasts through the Wnt signaling pathway. Alginate-Nanohydroxyapatite-Collagen (Alg-nHA-Col) microspheres have been shown to provide a good osteogenic microenvironment. The study reported that the viability, proliferation rate (8.5-fold increase vs. controls at 21 days) and mineralization ability of encapsulated human MG63 osteoblasts are significantly improved [54]. Cheng et al. prepared nano-hydroxyapatite (nHA)/chitosan (CS) composite microspheres with a uniform particle size distribution and an ECM-like nanofiber structure using microfluidic technology and direct alkali-induced gelation. By upregulating the expression of osteogenesis-related genes in mesenchymal stem cells through activating signaling pathways like Wnt/β-Catenin and RAS/MAPK, strontium ions (Sr^2+^) promote osteogenesis (Figure 4). However, excessive Sr^2+^ concentrations may be detrimental to the repair of bone defects. In vitro experiments showed that the above composites significantly increased ALP activity and upregulated the expression of osteogenesis-related genes, such as Runx2, through the Wnt pathway [55]. Moreover, they both accelerated the repair of the defect in the rat cranial defect model. These findings indicate that strontium-based bioactive coatings boost osteogenesis both in vitro and in vivo, showing great potential in promoting bone regeneration.

#### 3.1.3. Drug Delivery

The use of carrier systems for drug delivery is also an important strategy in current bone tissue engineering. Bone morphogenetic proteins (BMPs) are a key family of growth factors involved in bone regeneration. They promote osteoblast differentiation and bone matrix mineralization, and play an essential role in skeletal development and homeostasis [56]. However, due to the supraphysiological doses required for BMP products and their side effects, the clinical application of BMPs is limited to a few specific indications. Therefore, the development of effective delivery systems for BMPs is of considerable importance in bone defect repair.

A dual-factor controlled release system composed of nanofibrous PLLA scaffolds and PLGA nanospheres has been constructed. BMP-7 and FGF-2 were encapsulated into fast-release and slow-release nanoparticles respectively to tune the release of the two factors. The scaffolds can synergistically promote the proliferation and differentiation of BMSCs in vitro and in vivo, thereby facilitating bone regeneration [57]. Another dual-factor delivery system based on hydroxyapatite microcarriers (HA-MC) enabled the combined delivery of osteogenic factor (BMP-2) and angiogenic factor (VEGF), providing a feasible strategy for bone regeneration through regulation of carrier size and factor release [58].

In addition, osteoclast inhibition is another important approach for enhancing bone regeneration. Since the differentiation, resorption and metabolism of osteoclasts all depend on an acidic microenvironment, constructing a pH-sensitive drug delivery system can be an effective strategy to reduce excessive osteoclast activity and improve osteogenesis. As a pH-sensitive gated nano-drug delivery system, CS@MSNs-Naringin can efficiently inhibit osteoclast activity and promote osteoclast apoptosis, while upregulating BMP-2 expression, ultimately promoting bone regeneration. Although the current research is still limited to the regulation of osteoclasts by drug-loaded nanoparticles in in vitro and in vivo experiments, this delivery system represents a promising preclinical strategy for metabolism-related bone diseases such as osteoporosis [59].

Overall, not all nanomaterial strategies discussed for bone repair are equally mature from a translational perspective. Bioactive systems, especially nanohydroxyapatite-based coatings and osteoconductive scaffolds with simpler compositions, seem to hold stronger clinical promise. Their localized function, bone-mimetic composition, and implant-based application scenarios are well aligned with existing orthopedic practice. In contrast, multifunctional platforms such as 4D-printed or stimulus-responsive constructs are conceptually attractive because they may better adapt to irregular defects and dynamic healing environments. However, their material and manufacturing complexity also creates uncertainty. Likewise, growth factor delivery systems offer important biological advantages, yet unresolved issues remain in dose control, release predictability, and safety, particularly for potent osteogenic cues such as BMPs. Notably, bone tissue engineering challenges go beyond osteogenic capacity, involving scalable production, large-defect verification, interfacial durability, and clinically grounded benefit–risk evaluation of nanocomposites.

### 3.2. Skin Regeneration

**Figure 5:**
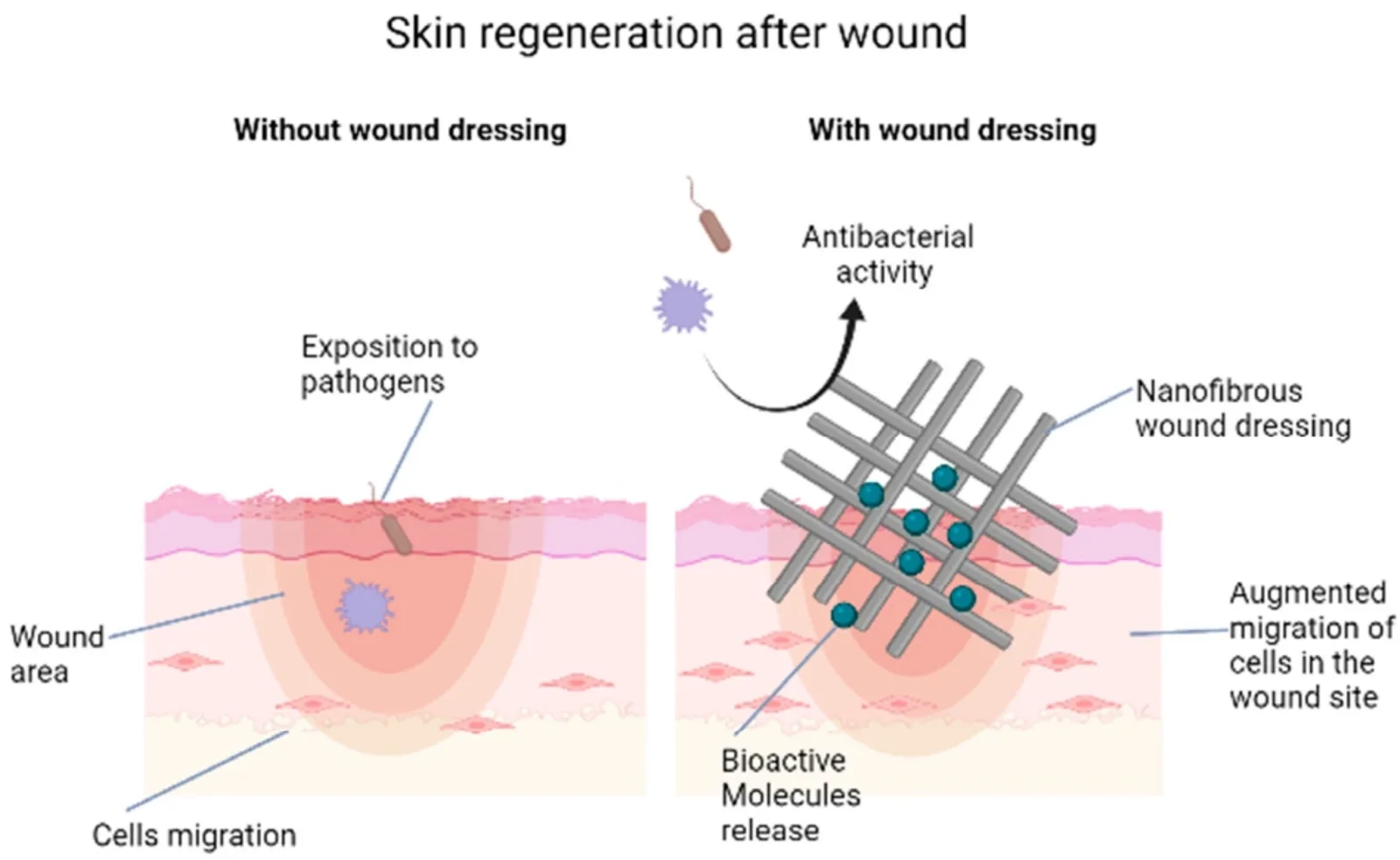
Schematic representation of the role of nanofibrous wound dressing in skin wound regeneration. Compared with untreated wounds, nanofibrous wound dressings can protect the wound area from pathogen exposure, provide antibacterial activity, release bioactive molecules, and enhance cell migration at the wound site, thus facilitating skin regeneration. Reprinted with permission from Ref. [61].

Skin is the largest organ of the human body and serves as the first defensive barrier. However, it is highly susceptible to mechanical injury, burns, diabetic ulcers, and other pathological conditions. Also, its healing process is easily influenced by the pathological microenvironment. Large or chronic skin wounds therefore remain a major clinical challenge and are frequently associated with limited donor availability, delayed healing, and postoperative scarring [60]. In recent years, the rapid development of nanomaterials has enabled a range of innovative therapeutic strategies for skin repair. Nanomaterials with antibacterial, anti-inflammatory and antioxidant properties, as well as adaptive multifunctional composites, represent an appropriate formulation to enhance the efficiency of wound repair. They are expected to treat different types of skin wounds and enhance the success rate of skin regeneration (Figure 5) [61].

#### 3.2.1. Antibacterial Nanofibers

Antibacterial activity is an important prerequisite for wound healing and skin regeneration. By eliminating pathogenic bacteria and reducing excessive inflammation at the wound site, nanomaterials can help establish a relatively stable and sterile microenvironment that is conducive to tissue repair. Among them, ZnO nanoparticles have attracted attention because of their better antibacterial and anti-inflammatory effects. Studies have shown that ZnO NPs can generate ROS to damage bacterial DNA, proteins and lipids. They can also release zinc ions which directly enter the cell membrane to interfere with bacterial metabolism, and damage bacterial cell membranes through physical interactions. Bactericidal effects and the ability to regulate inflammatory responses coexist in these nanomaterials. Experimental results have demonstrated that ZnO NPs exhibit significant antibacterial activity against *S. aureus*, and that this activity increases with the elevation of ZnO NP concentration [36].

In addition, ZnO/CuO-Cs nanocomposite dressings prepared by green synthesis have shown strong antibacterial performance. This material exhibits high porosity, good hydrophilicity and mechanical stability, absorbing exudate while maintaining a moist healing microenvironment. It destroys bacterial membranes and inhibits metabolism through the synergistic release of Zn^2+^ and Cu^2+^. Meanwhile the porous structure enhances antibacterial efficacy. In vitro experiments reveal that it eliminates *E. coli* within 3 h and inactivates *S. aureus*/MRSA within 24 h. Exhibiting potent bactericidal effects against Gram-positive, Gram-negative, and drug-resistant bacteria, this material holds great translational potential for wound dressing applications [62].

#### 3.2.2. Vascularization Accelerators

Nanomaterials also act as accelerators for angiogenesis. Vascular regeneration plays an irreplaceable role in wound healing by ensuring nutrient supply, microenvironment regulation, signal molecule transport, and skin function reconstruction. Appropriate angiogenesis is the hub for regulating scar formation and a key to chronic wound healing. The essence of angiogenesis lies in the endothelial cell proliferation and the formation of new blood vessels [63].

One representative example is a nanozyme constructed by the in situ growth of ultrasmall copper sulfide clusters on the surface of a nanofibrillar lysozyme assembly (NFLA/CuS NHs). This nanozyme functions as an alternative to nitrite reductase and can temporally regulate nitric oxide (NO) concentration to achieve both antibacterial and pro-angiogenesis effects. A high NO concentration is generated at the early stage to ensure bactericidal activity. Subsequently, a sustained release of lower NO concentration at the later stage promotes the migration of vascular endothelial cells and angiogenesis. In vitro experiments demonstrated that low concentrations of NO catalytically released by NFLA/CuS NHs could directly activate vascular endothelial cell migration. And a moderate concentration of NO fosters angiogenesis. In an in vivo MRSA-infected wound model, this material upregulated the expression of angiogenic markers (VEGF, CD31), promoted vascularization, and simultaneously improved nutrients and oxygen supply to the wound site. However, the concentration of NO must be carefully controlled, as excessively high NO levels may impede the repair process [64].

#### 3.2.3. Adaptive Dressings

Skin healing is a highly dynamic and complex process involving coordinated cellular and molecular mechanisms. From the initial hemostatic response to the subsequent proliferative phase, wound healing consists of four stages: hemostasis, inflammation, proliferation, and remodeling [60,61]. In contrast to conventional materials which mainly provide a moist environment and passive coverage, adaptive dressings offer a more responsive strategy for wound management. Self-adaptive dressings can transform their structure and function by sensing signals in the wound microenvironment, such as pH, ROS, glucose, and enzyme activity, to match the healing phases [65].

Multiple crosslinked, self-healing, and shape-adaptable hydrogel laden with pain-relieving chitosan@borneol nanoparticles (PGSB) exhibit multiple functions including physical barrier, pH-responsive drug release, antibacterial, anti-inflammatory, analgesic, and pro-angiogenic activities, representing a typical multifunctional wound dressing. PGSB exhibits synergistic physicochemical crosslinks based on dynamic boronic ester bonds, Zn^2+^ coordination by PVA-GA and SA-PBA, and hydrogen bonding, endowing it with adhesiveness, injectability, self-healing, and shape adaptability. These features allow the material to conform to irregular and dynamic wound sites and thus support more effective repair. In an in vivo burn model infected with *S. aureus*, the PGSB group achieved the optimal wound closure rate (99.1% after 14 days), along with the best healing effects and the lowest infection levels. It is acknowledged that adaptive dressings can overcome the limitation of conventional wound dressings and may be particularly valuable for the healing of dynamic wounds [66].

These studies highlight the value of nanomaterials in orchestrating multiple phases of skin repair, from infection control and inflammation modulation to angiogenesis and tissue reconstruction. From a translational standpoint, nanomaterial-based strategies for skin regeneration may be more readily implemented than those targeting deep tissues because the wound site is directly accessible and treatment is typically localized. Within this section, antibacterial nanofibers and wound dressings appear particularly promising because infection control and moisture management are already established clinical priorities, and these systems can often be incorporated into familiar dressing formats. By comparison, angiogenesis-regulating nanozymes and highly adaptive multifunctional dressings provide broader biological control over chronic or infected wounds, but their translational advantage is less straightforward when the formulation complexity increases. In addition, debates continue regarding the safe therapeutic window of metal-ion and nitric oxide release. Insufficient release may limit efficacy, while excessive local exposure may impair tissue repair or increase toxicity. Nevertheless, even with encouraging preclinical data, barriers including long-term biosafety, degradation characteristics, chronic wound assessment, and cost-efficient fabrication need to be resolved before multifunctional skin-regenerative nanoplatforms can be considered clinically mature.

Future studies should focus on the development of degradable and functionally integrated nanocomposite systems tailored to different wound types. In combination with advanced fabrication approaches such as 3D printing and biomimetic tissue engineering, these strategies may facilitate the construction of smarter and more clinically relevant platforms for skin regeneration [67].

### 3.3. Neural Tissue Engineering

Beyond applications in bone and skin repair, nanomaterials have also opened new possibilities for neural tissue engineering. As the structural and functional basis of the nervous system, neural tissue is essential for sensation, movement, and regulation. However, brain injury, spinal cord injury, neurodegenerative diseases, and other harmful parameters can cause neuronal death, axonal rupture, and other damage, severely impairing the function of the nervous system and making repair extremely difficult [22]. But nanomaterials offer new opportunities. Their unique physicochemical properties, especially tunable electrical conductivity and surface function, enable precise regulation of neural cell behavior, including cell adhesion, neurite outgrowth, and functional recovery. This section examines how nanomaterials are reshaping strategies for both peripheral and central nerve repair.

#### 3.3.1. Electroactive Scaffolds

Nervous tissue is inherently electroactive. Neurons rely on electrical signals to transmit information, and axon growth is highly dependent on the electrical microenvironment. Conventional polymer scaffolds are generally insulating and cannot adequately mimic the electrical properties of native nerves. In contrast, conductive nanomaterials can provide electrical cues that significantly promote and regulate neural regeneration [68,69].

Among these materials, conductive nanocomposite hydrogels (CNHs) have stood out due to their high water content, tunable mechanical properties, and excellent electrical conductivity. By incorporating carbon-based nanomaterials, metal-based nanomaterials, conductive polymer, and piezoelectric nanofillers, CNHs can accurately mimic the electroactive microenvironment of neural tissue and support biomimetic electrical signal transmission. When further combined with nanotopographical structures and bioactive cues, these systems can effectively promote neuronal differentiation, axonal growth, and myelination. For central nervous system (CNS) repair, CNHs often integrate antioxidant, immunomodulatory, and wireless electrical stimulation functions to alleviate oxidative stress and neuroinflammation after spinal cord injury and traumatic brain injury. For peripheral nervous system (PNS) regeneration, CNHs significantly enhance axon regeneration and functional recovery through directional topography, piezoelectric response, and magnetic guidance. Nevertheless, unclear long-term biosafety, a lack of humanized models, and an imperfect standardized evaluation system continue to hinder their clinical translation [22].

#### 3.3.2. Guidance Conduits

Nerve guidance conduits (NGCs) have attracted extensive attention and emerged as a promising strategy. These tubular bioscaffolds are designed to bridge nerve defects and provide a supportive microenvironment for regeneration. By guiding the directional extension of nerve fibers and limiting scar tissue infiltration, NGCs are considered a potential alternative to autologous nerve grafts [70].

Xu et al. developed an ultrasound-responsive aligned piezoelectric nanofiber which features bilayer structure. The inner layer consisted of barium titanate piezoelectric nanoparticles (BTNPs)-doped polyvinylidene fluoride-trifluoroethylene [BTNPs/P(VDF-TrFE)] electrospun nanofibers, which converted ultrasonic stimulation into non-invasive wireless electrical stimulation and induced directional neural growth via topological guidance. The outer layer was a thermoresponsive hybrid hydrogel that enabled ultrasound-triggered controlled release of nerve growth factor (NGF). Ascribed to these beneficial features, both in vivo and in vitro studies demonstrated that the NGC could positively enhance the axonal regeneration and function recovery of sciatic nerve defects [18].

The GelMA/PLys@PDA-Fe@PLCL composite nerve guidance conduit which consists of aligned PLCL nanofibers modified with PDA-Fe^3+^ and PLys with aligned GelMA hydrogel also exhibited stable mechanical properties and good hydrophilicity. In addition to promoting the oriented growth of Schwann cells, it can regulate macrophage polarization and exert antioxidant and anti-inflammatory effects [71]. Owing to their unique ability to promote nerve regeneration, a variety of different nerve conduit materials, such as nanofiber-based responsive sponge scaffolds and shape-persistent conductive nerve guidance conduits, have been fabricated and their potential for nerve repair and related biomedical applications are highlighted [72,73]. The diversity in NGC design reflects the complex requirements of nerve repair and underscores their potential in neural tissue engineering.

#### 3.3.3. Growth Factor Release Systems

**Figure 6:**
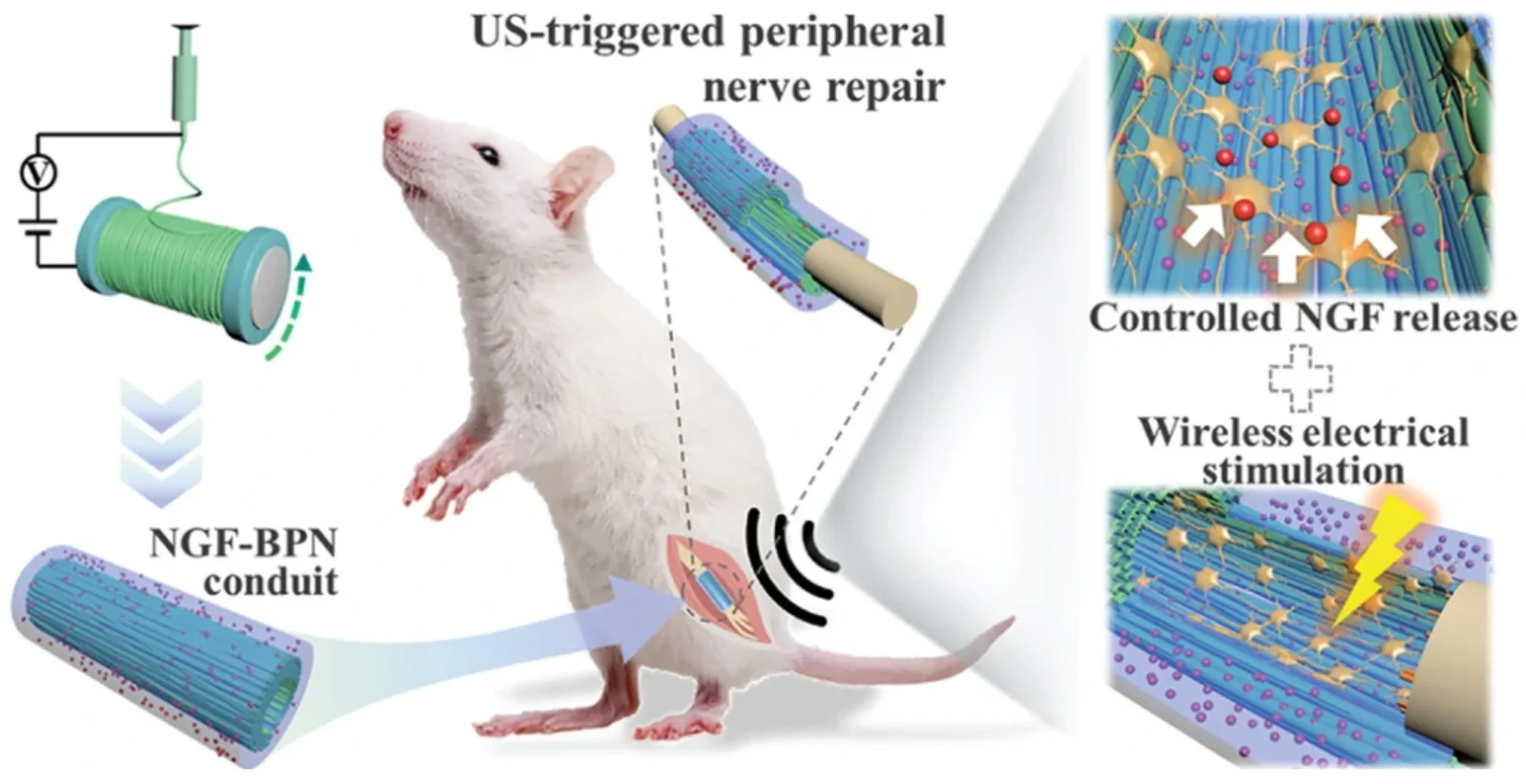
Schematic illustration of the ultrasound-responsive NGF-BPN hydrogel conduit for peripheral nerve regeneration. Upon ultrasound stimulation, the conduit facilitates controlled NGF release and wireless electrical stimulation, synergistically enhancing peripheral nerve repair. Reprinted with permission from Ref. [18].

A wide range of growth factors is involved in nerve regeneration, and nanomaterials capable of loading and controllably releasing these bioactive molecules, which have shown considerable promise for neural repair [74]. In particular, nanosystems that control the release of neurotrophic factors indicate great potential in facilitating neuroregeneration (Figure 6) [66].

The poly(2methacryloyloxyethyl phosphorylgallylcholine) (PMPC)/L-arginine (PMPC/A)-based nanomotors which are loaded with the NO synthase inhibitor 1400 W and nerve growth factor (NGF) can penetrate the blood-spinal cord barrier (BSCB) and target the nerve injury sites. The nanomotor enables sustained and slow release of NGF and thus promotes neuronal repair [75]. Similarly, loading brain-derived neurotrophic factor (BDNF) onto electrospun nanofibrous scaffolds through PDA surface modification allows for the controlled release of BDNF. Release profiles showed that approximately 75% of BDNF was released from the scaffold at a relatively constant rate over 28 days, achieving long-term sustained release [76].

To further improve the transport of neurotrophins across the blood–brain barrier (BBB), a nanocapsule-based system has also been developed to enable intravenously administered NGF to enter the CNS. By in situ polymerization, 2-methacryloyloxyethyl phosphorylcholine (MPC) and polylactic acid (PLA) diacrylate are enriched around the NGF and form the nanocapsules. Encapsulation allows NGF to cross the BBB, while subsequent hydrolysis of the crosslinkers leads to disintegration of the polymer shell and sustained release of NGF. The biocompatibility and therapeutic efficacy of this strategy have been validated in both mouse and non-human primate models [77]. With the help of nerve growth factors, it will be possible for nerve to restore to the pre-injury state.

In summary, nanomaterial-based neural repair strategies are expanding the therapeutic scope of neural tissue engineering because they can address several key barriers to neural regeneration simultaneously, including the lack of electrical cues, insufficient directional guidance, and poor local retention of trophic factors. Among the currently explored approaches, conductive nanocomposite hydrogels and nanofiber-based nerve guidance conduits appear particularly attractive. However, their progress toward clinical translation varies greatly. For central nervous system applications, chronic neuroinflammation, degradation control, and the difficulty of achieving meaningful functional recovery in injury models remain major obstacles. For peripheral nerve repair, guidance conduits may be closer to translation than more complex nanosystems. In addition, contradictory findings regarding the biocompatibility of conductive nanomaterials, particularly carbon-based systems, often arise from differences in composition, purity, dose, and implantation context. Therefore, future progress in neural tissue engineering will depend less on adding material complexity and more on establishing standardized safety evaluation and robust evidence of long-term functional benefit.

### 3.4. Cardiac Tissue Engineering

Cardiac tissue repair remains particularly challenging because myocardial tissue has limited regenerative capacity. Following myocardial infarction and other forms of cardiac injury, the loss of viable cardiomyocytes is often accompanied by disrupted electrical conduction, poor vascular supply, oxidative stress, and prolonged inflammation. These parameters lead to adverse remodeling and functional decline. For this reason, considerable attention has been directed toward material systems that can simultaneously support structural repair and modulate the injury microenvironment. Recent work suggests that nanomaterials are well suited to this task, as their physicochemical tunability allows for the incorporation of conductive, pro-angiogenic, and immunoregulatory functions within a single platform.

#### 3.4.1. Electroconductive Composites

Conductive nanomaterials have emerged as a pivotal platform in cardiac tissue engineering, which offer mechanical support and restored electrical communication for regenerating cardiac function [78,79]. Carbon-based nanomaterials are widely used to render hydrogels conductive. Functionalized CNTs can be integrated into polymer matrices such as gelatin methacrylate (GelMA) and collagen. They boosted electrical conductivity and mechanical strength while enhancing cardiomyocyte alignment, intercellular electrical coupling, and the expression of maturation-related markers. Similarly, graphene and reduced graphene oxide (rGO) can be integrated into hydrogel networks through blending or in situ reduction. These conductive systems supported electrical signal propagation, angiogenesis, and the delivery of therapeutic cells or genes to improve cardiac function after myocardial infarction (MI). Although these nanomaterials offer great opportunities for developing functional conductive hydrogels, their animal studies are not sufficient for their clinical translation [80].

Beyond carbon-based systems, emerging two-dimensional nanomaterials have further expanded the design space of conductive cardiac scaffolds. Using aerosol jet 3D printing, Ti_3_C_2_T_x_ MXene can be patterned onto PEG hydrogels. The Ti3C2Tx MXene-PEG composite constructs can mimic the ordered structure of ECM and the electroconductivity of the human heart, therefore upregulating cardiac maturation genes such as MYH7 and guiding the oriented growth and synchronous beating of human induced pluripotent stem cell-derived cardiomyocytes (hiPSC-CMs) [42]. Designed as cardiac patches or injectable hydrogels, such nanocomposite systems can improve matrix conductivity, optimize electrical signal transmission, and ultimately support myocardial regeneration. Undoubtedly, electroconductive scaffolds are addressing the lack of electrical conductivity in traditional materials.

#### 3.4.2. Angiogenic Nanoplatforms

**Figure 7:**
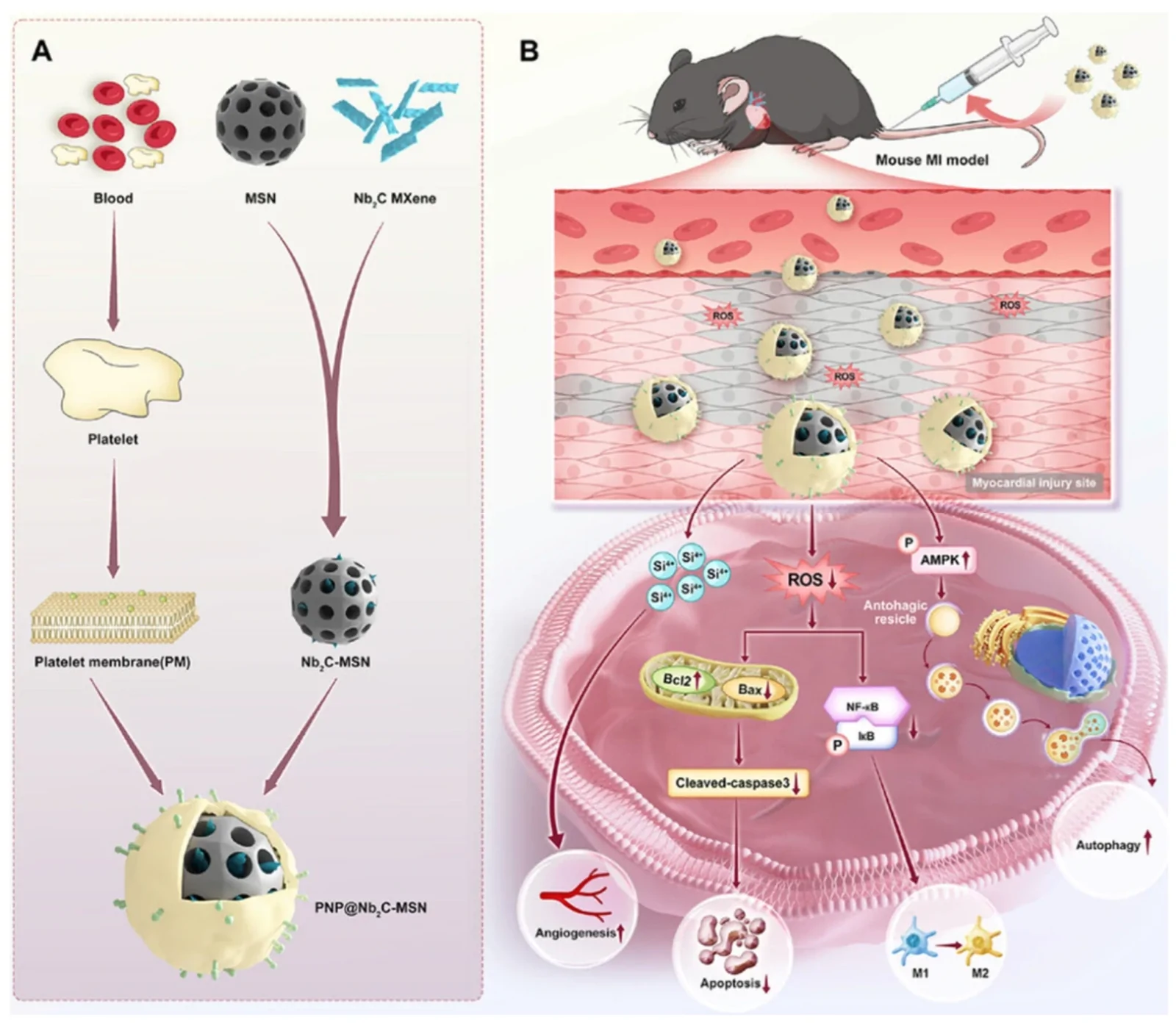
Schematic illustration of the preparation of PNP@Nb_2_C-MSN and its proposed mechanism for myocardial infarction treatment. The platelet membrane-coated nanoplatform enables targeted delivery to the infarcted myocardium, where it scavenges ROS, suppresses apoptosis and inflammation, and enhances angiogenesis and autophagy, thereby promoting cardiac tissue repair. Reprinted with permission from Ref. [66].

Early revascularization to restore blood supply to the myocardium is crucial in treating heart diseases. Angiogenic nanoplatforms offer effective strategies to enhance revascularization and improve cardiac function in ischemic myocardial repair. Yang et al. developed a biomimetic PNP@Nb_2_C-MSN nanoparticle comprising Nb_2_C MXene, mesoporous silica, and platelet membrane coating, which promoted angiogenesis and enhanced autophagy through AMPK signaling (Figure 7). In vivo experiments further validated that PNP@Nb2C-MSN facilitates angiogenesis within the infarcted region and improves blood supply. This composite serves as a promising approach for the treatment of ischemic injuries [81]. Another SDF-encapsulated lipid nanoparticles that selectively accumulated in ischemic myocardium can effectively recruit endothelial progenitor cells, activate the PI3K/Akt pathway, and improve capillary density and microvascular perfusion [82].

Nitric oxide (NO) is also considered as an ideal pro-angiogenic factor at the appropriate concentration. The peroxynitrite-free NO-embedded nanoparticles (OFEN) enabled long-term NO delivery and maintained a stable concentration of NO which was suitable for angiogenesis. Meanwhile, OFEN can convert ROS into O_2_ and inhibit NO from converting into harmful peroxynitrite (ONOO–) in MI tissues. Through HIF-1α signaling, these nanoparticles promoted both angiogenesis and arteriogenesis, thereby improving perfusion and reducing fibrosis and apoptosis [83].

#### 3.4.3. Anti-Inflammatory Systems

Besides electrical dysfunction and vascular insufficiency, excessive inflammation is another major barrier to effective cardiac repair. The inflammatory cascade triggered after myocardial injury can amplify oxidative stress, aggravate tissue damage, and interfere with constructive remodeling. This has prompted increasing interest in nanomaterial-based systems capable of combining antioxidative activity with immune regulation.

The biomimetic PNP@Nb_2_C-MSN nanozyme mentioned above not only promotes angiogenesis, but also scavenges reactive oxygen species (ROS), inhibits NF-κB-mediated inflammation, and promotes M2 macrophage polarization to alleviate myocardial injury [81]. Chen et al. developed P-MSN/miR-199a-5p nanoparticles for targeted delivery of miR-199a-5p, which suppressed oxidative stress and inflammatory injury by regulating AGTR1 and improved cardiac contractility via MARK4 [84]. Yang et al. fabricated an injectable ROS-responsive hydrogel SMM@Gel carrying macrophage-targeting danshensu sodium nanoparticles, which enabled sustained drug release and ROS scavenging. By attenuating inflammation, promoting M2 polarization and enhancing angiogenesis, it improved the myocardial microenvironment for myocardial infarction treatment [85]. These findings have indicated that anti-inflammatory nanosystems may contribute to cardiac repair not only by reducing secondary injury but also by creating conditions more favorable for tissue regeneration.

The studies discussed above reflect a clear shift in cardiac tissue engineering from single-function materials toward platforms able to intervene in several aspects of infarction remodeling. Conductive composites hold particular appeal for cardiac repair, as impaired electrical coupling represents a hallmark of myocardial damage. However, conductive benefit alone is unlikely to be sufficient unless electrical safety, biodegradation behavior, and stable integration with host myocardium are ensured. Angiogenic and immunoregulatory nanosystems further expand the therapeutic scope by targeting vascular insufficiency and inflammatory injury, but their in vivo distribution and retention remain difficult to control. Many reported systems combine several functions within a single platform, which improves preclinical performance but also complicates manufacturing and mechanistic interpretation. Nevertheless, while animal study data show encouraging results, cardiac tissue engineering still confronts multiple translational obstacles. It remains critical to achieve reliable myocardial delivery, ensure biosafety, and verify whether multifunctional nanoplatforms can generate sustained functional benefits beyond short-term improvements in surrogate markers.

Although functional nanomaterials are often discussed according to tissue type or formulation strategy, their translational value is better understood through both material class and dominant regenerative mechanism (Table 1). Across the studies reviewed here, major nanomaterial platforms include nanoparticles, nanofibers, MXene-based materials, nanocrystalline mineral systems, conductive materials, and liposomal or biomimetic delivery systems. Each of them offers distinct advantages, but they do not contribute to tissue repair equally. In general, scaffolds such as nanofibers, hydrogels, and mineralized composites are particularly useful for recreating extracellular architecture and providing physical guidance. Nanoparticle- and liposome-based systems are more effective for drug delivery, microenvironment modulation, and controlled intervention. Conductive materials, including MXenes and carbon-based nanomaterials, are especially attractive for electroactive tissues, while bioactive mineral nanomaterials confer superior benefits to bone repair because of their chemical similarity to native hard tissue.

Despite the diversity of nanomaterials, several regenerative mechanisms recur across different tissue systems, including ROS regulation, macrophage polarization, angiogenesis, antibacterial activity, electrical stimulation, topographical guidance, and controlled release of growth factors. For example, ROS scavenging and inflammation control are relevant in skin wounds, neural injury, and myocardial infarction, while angiogenesis is essential for both wound healing and ischemic tissue repair. In comparison, other mechanisms have higher tissue specificity. Mineralization and osteoinductive signaling are central to bone regeneration, electrical coupling is important in neural and cardiac repair, and adaptive wound-interface management plays a key role in skin regeneration. These distinctions are important because they clearly suggest that translational priorities should be defined not only by the novelty of a nanomaterial platform, but by how effectively its functions address the biological bottlenecks of the target tissue.

## 4. Advanced Fabrication Technologies

### 4.1. High-Precision Manufacturing Methods

**Figure 8:**
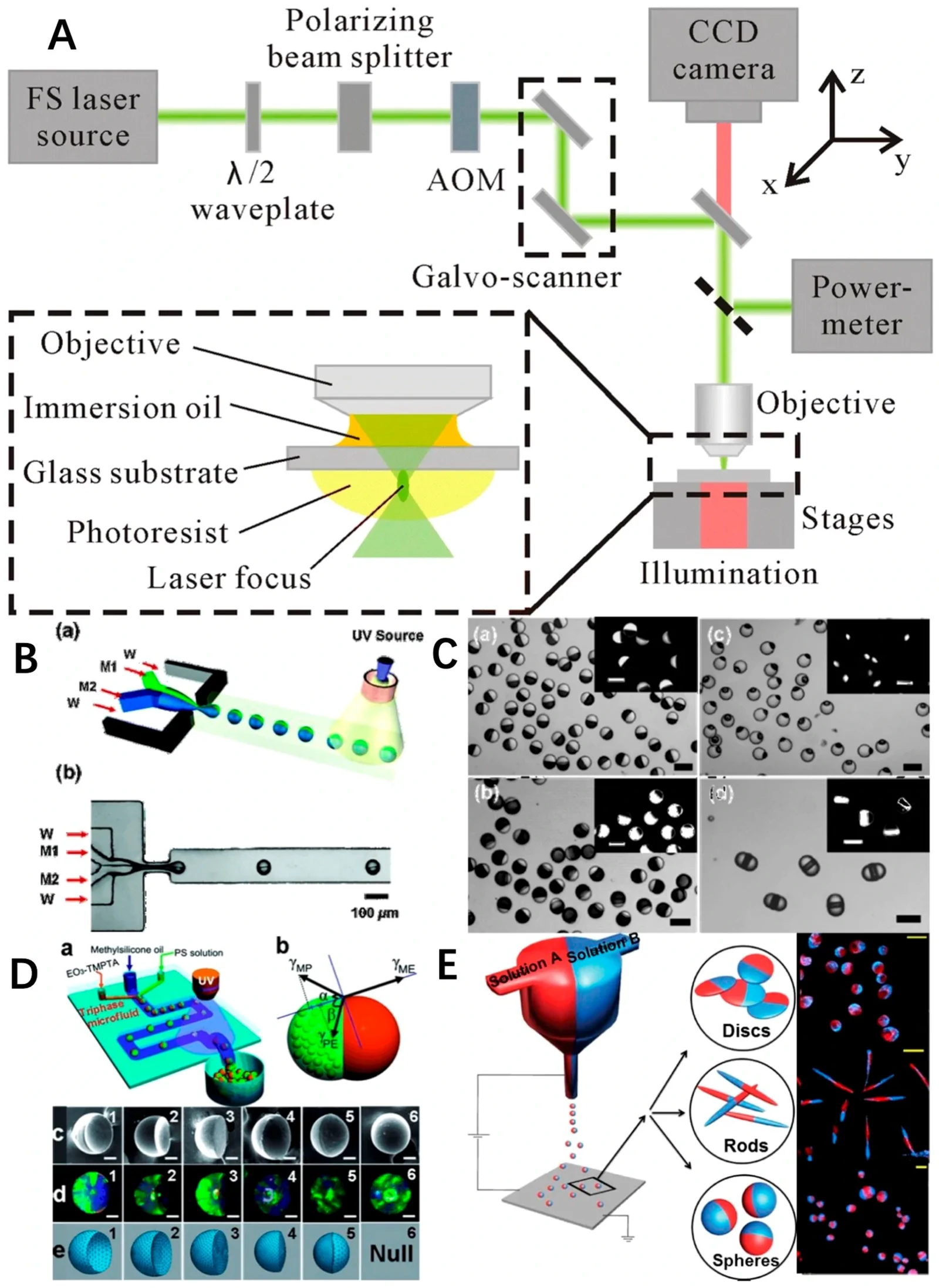
Schematic illustrations and representative experimental results of 2PP fabrication and microfluidic/Janus structure preparation strategies. (**A**) Schematic diagram of 2PP fabrication system. (**B**) Microfluidic preparation strategy for polymeric Janus particles (JNPs): (**a**) three-dimensional schematic of the microfluidic synthesis process; (**b**) top-view layout of the microfluidic chip with inlet streams M1, M2, and W. (M1: Methacryloxypropyl dimethylsiloxane; M2: acrylic acid (5.0 wt %), pentaerythritol triacrylate (45.0 wt %), and poly(ethylene glycol) diacrylate (45.0 wt %); W: SDS). (**C**) Representative optical microscopy images of M1-M2 Janus particles and ternary structures obtained at different flow rates of M1, M2, and W: (**a**) 0.02, 0.02, 1.5; (**b**) 0.01, 0.03, 1.5; (**c**) 0.04, 0.008, 2; and (**d**) 0.14, 0.16, 10. (mL/h) (**D**) Schematic illustration of the formation of Janus structures: (**a**) schematic of the microfluidic device and the three fluid phases; (**b**) geometric model of interfacial tensions and characteristic angles; (**c**–**e**) evolution of particle morphology and internal structure during formation, presented as grayscale images, fluorescence images, and 3D renderings, respectively. (**E**) Electrohydrodynamic cojetting strategy for the preparation of various Janus structures composed of PS, EO3-TMPTA, and methylsilicone oil. Adapted with permission from Refs. [86,87].

A defining objective of tissue engineering is to recreate the structural and biochemical features of the native microenvironment and direct cell behavior in a predictable manner. This requirement places stringent demands on the design and fabrication of nanomaterials, which must possess precise architectures, controllable physicochemical properties, reliable biocompatibility, and integrated biological functions. In this context, advanced manufacturing technologies have become essential for the preparation of next-generation nanomaterials. Among the rapidly evolving approaches, two-photon polymerization, microfluidic synthesis, electrospinning, and self-assembly have emerged as particularly important tools. These state-of-the-art methods offer levels of precision and tunability that are difficult to achieve using conventional fabrication methods (Figure 8).

#### 4.1.1. Two-Photon Polymerization for Sub-100 nm Resolution Scaffolds

Due to the ability of 3D scaffolds to provide a biomimetic microenvironment for cell adhesion, proliferation, and differentiation, the fabrication of high-precision 3D scaffolds has become a research hotspot. Conventional fabrication methods, however, are often constrained by limited resolution and geometric inflexibility, which restrict their ability to produce well-defined nanostructures. Based on two-photon absorption (TPA), two-photon polymerization (TPP) enables fabrication at resolution beyond the diffraction limit. Furthermore, the probability of two-photon absorption is proportional to the square of the laser intensity, resulting in significant polymerization only in the focal region of the laser. Finally, by controlling the movement of the laser focal spot, layers are stacked to form complete three-dimensional nanoscaffolds. In this way, TPP can exceed the diffraction limit associated with traditional single-photon absorption, improve resolution to the nanometer scale, and achieve truly 3D arbitrary structure writing [88,89].

This unique writing mechanism has enabled TPP to achieve nanoscale resolution and true 3D freeform fabrication, making it particularly suitable for constructing scaffolds with finely defined micro- and nanostructures. Studies have shown that TPP can produce sub-100 nm features, with gap sizes below 100 nm, which markedly exceeds the resolution of most conventional 3D printing techniques [86,90]. But due to the high technical threshold of TPP, its manufacturing scale is limited. On the one hand, some metal nanoparticles exhibit low loading capacity in TPP photoresists, making it difficult to integrate nanofunctional units [91]. On the other hand, the nanostructure of scaffolds is overly complex, and the production cannot ensure the uniformity of performance and quality [88]. But using TPP to achieve high-precision 3D printing of nanomaterials remains a promising research direction.

#### 4.1.2. Microfluidic Synthesis of Janus Nanoparticles for Dual-Targeting Delivery

Over the last decades, searching for novel smart nanomaterials with tailored qualities and desired capabilities has sparked a surge of interest. Among them, Janus nanoparticles (JNPs) have attracted particular interest. JNPs are anisotropic nanoparticles composed of two distinct sides with different chemical nature and polarity on each side, which resemble a “double-faced structure”. With their interesting characteristics, JNPs have multiple functions such as targeted delivery, bioimaging and treating cancers.

Traditional synthesis methods for JNPs, such as masking, phase separation and self-assembly, suffer from drawbacks including complicated procedures, cumbersome operations, and uneven size distribution. Owing to its core advantage of precisely controlling reaction conditions, the microfluidic approach has gradually become a mainstream strategy for JNP synthesis [92,93,94]. The core strategies of microfluidic technology include co-flowing, phase separation, double emulsion and electrohydrodynamic co-jetting. Through accurate regulation of fluid behavior in microchannels, these methods have enabled the production of monodisperse JNPs with narrow size distributions while also allowing flexible adjustment of particle morphology and functional attributes.

Despite these advantages, several technical barriers have persisted. For instance, the co-flow method requires matching the viscosity of the dispersed phase, while the double emulsion method imposes extremely high requirements on flow rate control [95]. With the iterative upgrading of technology in the future, it is expected that microfluidic methods will promote the large-scale production and commercialization of JNPs, providing better solutions for targeted delivery, precision therapy and other applications.

Methods represented by TPP and microfluidic technology provide better solutions for the precise fabrication of nanomaterials. From the precise synthesis of nanoparticles to fabricating 3D nanoscaffolds, these advanced synthesis techniques endow nanomaterials with multi-scale and high-precision characteristics, realize the regulation of 3D structures and micro-interfaces of nanomaterials, offering key technical support for the preparation of nanomaterials.

### 4.2. AI-Assisted Design and Fabrication

Recent years have witnessed a spurt of progress in AI technology, which has shown great potential in medicine, including the development of nanomaterials [96]. As the structural complexity and functional requirements of nanomaterials continue to increase, conventional trial-and-error strategies have become less efficient for their design and optimization. Therefore, AI-assisted approaches have emerged as valuable tools for improving the design, fabrication, and performance prediction of nanomaterials. Approaches such as machine learning and other computational methods can handle numerous parameters in nanoparticle synthesis, such as the nanoparticle synthesis and complex nano-bio interactions. They can model and reproduce the complex relationships, thereby assisting the rational design of nanoparticles [97,98]. These capabilities are creating new opportunities for the complex and data-driven development of nanoparticles, although the degree of experimental validation still varies considerably among different AI-based approaches.

#### 4.2.1. Generative Adversarial Networks (GANs) Predicting Nanoparticle-Cell Interaction Outcomes

The rapid development of neural networks and the application of adversarial ideas have led to the birth of GANs. Composed of a generator and a discriminator, GANs are inspired by two-player zero-sum game. GANs are typical adversarial learning frameworks, and the goal is to estimate the potential distribution of real data samples and generate new samples. Through this adversarial process, GANs can learn the underlying distribution of experimental data and generate new samples with similar characteristics. Owing to their strong generative and optimization capabilities, GANs have been explored as useful tools for predicting the outcomes of nanoparticle-cell and nanoparticle-tissue interactions [99].

This capability is particularly relevant in tumor-targeted nanomedicine, where the enhanced permeability and retention (EPR) effect has enabled nanoparticles to accumulate preferentially in tumor tissues. However, the distribution of quantum dots (QDs) is highly heterogeneous, and can be affected by the complex tumor-nano interactions, making it difficult to describe with traditional models. To address this issue, Tang et al. developed a Generative Adversarial Network for Distribution Analysis (GANDA) to describe and generate the QDs distribution pixels-to-pixels. It demonstrated the ability to investigate the tumor-nano interactions with high reliability and provided the intratumoral NPs distribution, which enabled virtual quantitative downstream analysis to improve the success rate of nanomedicine therapy.

Nevertheless, GAN-derived models are still in the preliminary developmental stage for relevant applications and face many challenges in precision and generalization. For example, the reported GANDA framework included only a single tumor model and was limited to predicting NP distribution in 2D images [100]. More importantly, several factors continue to restrict the wider application of GANs in nano medicine and tissue engineering. First, available training datasets are often limited in size and highly heterogeneous because nanoparticle behavior is influenced by multiple experimental variables. Second, GAN models generally exhibit poor interpretability, making it difficult to connect generated distribution patterns with biological mechanisms. Third, the complexity and dynamic nature of biological systems bring precision and generalization challenges to GANs. The heterogeneity of tissues makes it challenging for current GAN models to generalize from simplified 2D or single-model datasets to clinically relevant settings. Therefore, current GAN models are regarded as predictive approaches with future potential, rather than as fully validated experimental systems.

#### 4.2.2. Digital Twins for In Silico Optimization of Nanoparticle Behavior and Formulation Performance

Another emerging AI-assisted strategy is the digital twin, which serves as a real-time virtual replica of a nanomedicine system. By integrating experimental data and artificial intelligence algorithms, it has been developed to stimulate NPs interaction and predict pharmacokinetics. In contrast to GAN-based predictive models, digital twins have already shown partial experimental validation in specific nanomedicine settings. Especially for polymeric nanoparticles, the functions of digital twins have been experimentally validated. They can simulate nanoparticle interactions, predict particle size and drug release profiles, solving the problem of poor controllability in traditional synthesis and thereby achieving formulation optimization [101]. Beyond formulation control, the digital twin concept also holds particular relevance for tissue engineering, where scaffold degradation behavior, material-cell interactions, and therapeutic release profiles must be coordinated. Supported by early clinical studies, the construction of patient-specific digital twins for simulating personalized myocardial conduction helps elevate the accuracy of individualized cardiac tissue engineering [101].

Studies have also shown that digital twins have promising applications in nanomaterial assembly, immune digital twinning, and precision medicine. Nevertheless, these applications of digital twins remain more prospective and is still at an early stage of development [102,103,104]. At present, however, several challenges have continued to limit broader implementation. These include the difficulty of integrating heterogeneous datasets, the high computational demand required for accurate real-time simulation, and the challenge of building models that remain robust across diverse biological contexts. In the last few years, many studies have tried to point out the multiple challenges the scientific community faces to bring digital twins into the preclinical and clinical settings. Therefore, although a small fraction of digital twin models have been validated in preclinical experiments, most digital twin models still fail to bridge this gap and remain idealized digital models.

AI-assisted design and fabrication have introduced a shift from optimization toward predictive and data-driven engineering in the development of nanomaterials. However, the maturity of these approaches remains uneven. GAN models are currently most valuable as early-stage computational tools for identifying potential nanoparticle distribution patterns and nano-bio interaction trends, whereas certain digital twin applications, particularly in formulation optimization of polymeric nanoparticles, have already shown experimental validation. At the same time, broader applications in complex biological systems still require further validation, larger standardized datasets, and improved model robustness. As these obstacles are gradually resolved, AI-based strategies are expected to play an increasingly important role in connecting nanomaterial design with biological performance and translational application.

## 5. Clinical Translation Roadblocks & Solutions

**Figure 9:**
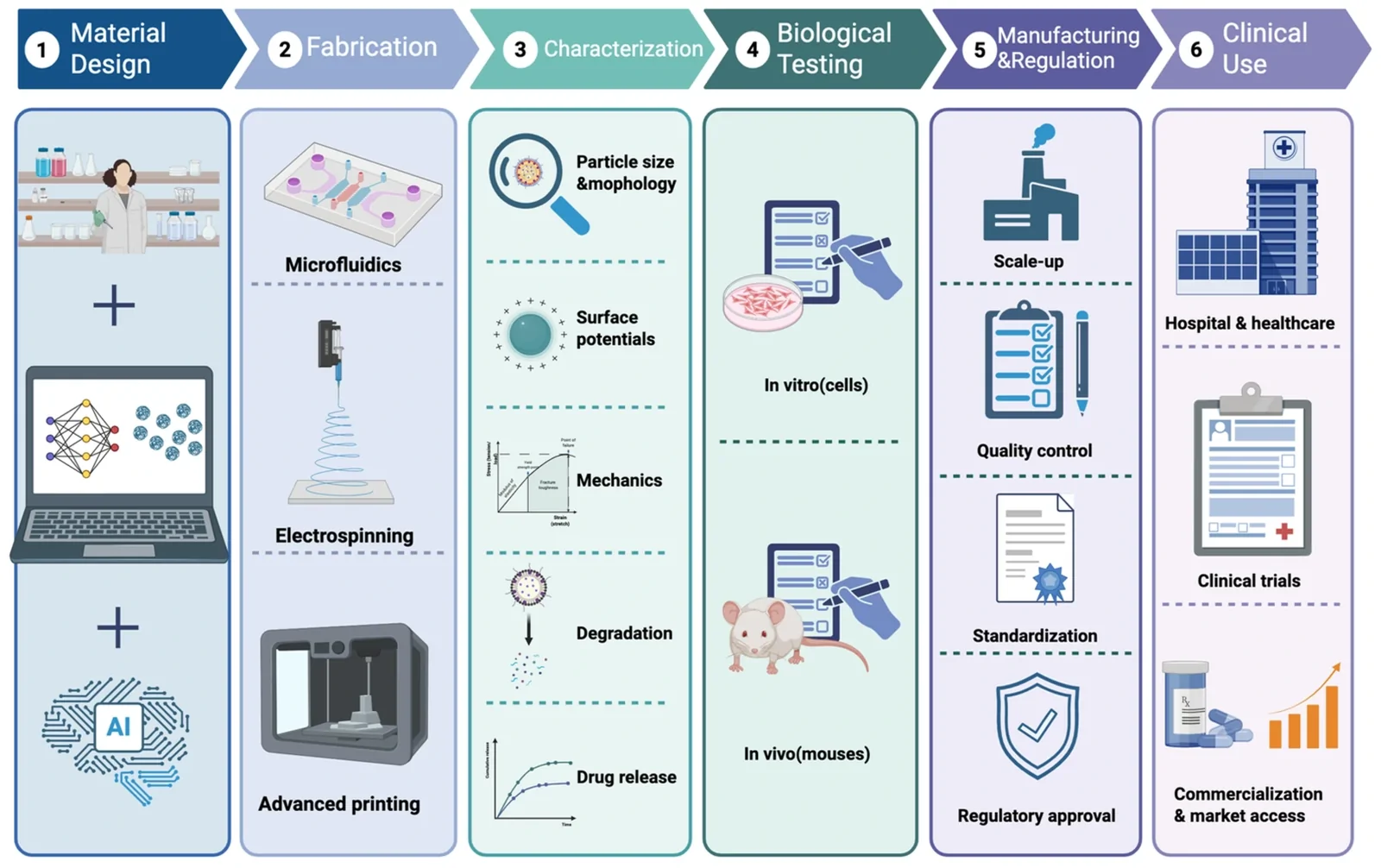
Schematic overview of the translational roadmap of functional nanomaterials from bench to bedside, covering material design, fabrication, characterization, biological testing, manufacturing and regulation, and clinical implementation.

Despite the rapid progress of nanomaterials and the continued emergence of advanced synthesis methods for various nanoparticles, numerous nanomaterials still encounter clinical translation barriers to varying degrees. Importantly, the limited progression of many nanomaterial systems beyond preclinical studies is not simply due to insufficient biological efficacy. In many cases, promising in vitro and small-animal results cannot be smoothly translated into clinical use because the final product must also satisfy requirements related to manufacturability, batch consistency, sterilization, storage stability, economic feasibility, and regulatory classification. Therefore, successful translation depends not only on material performance, but also on whether the nanomaterial can be reproducibly manufactured, safely stored, practically delivered, and evaluated through an appropriate approval pathway. A clear understanding of these roadblocks, together with corresponding solution-oriented strategies, is critical for advancing the clinical application of nanomaterials (Figure 9).

### 5.1. Biological Challenges

Among the factors influencing the clinical translation of nanomaterials, their intrinsic properties have remained particularly critical. These properties determine whether the materials can be successfully applied in diseases. The long-term cytotoxicity, immunogenicity, and uncontrollable in vivo behavior of nanomaterials are all major biological challenges in practical applications.

#### 5.1.1. Long-Term Nanotoxicity: Mitochondrial Dysfunction Induced by Persistent Nanoparticles (e.g., Quantum Dots)

Quantum dots are a class of nanocrystals with a diameter between 2 and 20 nm. They have excellent optical properties and nanoscale effects, so they have been widely used as fluorescent nanomaterials and have gradually become one of the indispensable materials in nanomedicine [105,106,107]. In general, QDs possess a large specific surface area and abundant surface-active sites, both of which strongly influence their biological behavior. For conventional QDs, toxicity has often originated from the release of metal ions and the spontaneous generation of ROS. As one of the most sensitive target organelles, mitochondria are particularly vulnerable to QDs. QDs can induce a decrease in mitochondrial membrane potential and swelling of mitochondria. ROS can also damage mitochondria, thereby triggering mitochondrial dysfunction and impairing cellular function [108].

A similar concern has been reported for black phosphorus quantum dots (BPQDs), which not only exhibited a strong capacity to promote ROS generation but also disrupted the cellular antioxidant system, thereby inducing a series of inflammatory responses. However, the toxicity of QDs was not absolute but was related to their surface modification degree and multifunctional shell layers. Naked and unmodified QDs such as BPQDs were highly prone to toxicity and inflammation, resulting in long-term nanotoxicity. High-quality QDs synthesized through surface modification and toxic element substitution, such as carbon quantum dots (CQDs) and graphene quantum dots (GQDs), have enabled the development of quantum dot materials with relatively low intrinsic toxicity for applications such as bioimaging [109].

#### 5.1.2. Immune Evasion Strategies: “Stealth” Coatings Using Phosphorylcholine-Based Polymers

In addition to direct cytotoxicity, the interaction between the physicochemical properties of nanomaterials and the biological environment can trigger complement activation and immune-cell recognition, thereby triggering inflammation, phagocytic clearance, and adaptive immune responses. These symptoms severely affect the efficiency and safety of nano-delivery systems. To mitigate such immune and inflammatory reactions, nanomaterials can be encapsulated in coatings to achieve immune evasion. Among these coatings, poly(2-methacryloyloxyethyl phosphorylcholine) (PMPC) stands out due to its unique biomimetic properties. Owing to the hydration of PMPC, it will not interact strongly with proteins or cells, thus exhibiting a better effect in alleviating immune activation [110].

Recent studies have suggested that modification of nanoliposomal drug carriers with hydrophilic coatings (PMPC-Lipo) can achieve efficient drug delivery while reducing inflammation. This material can not only restore the lubrication of damaged cartilage through hydration but also evade recognition by macrophages to avoid immune phagocytosis, thereby subtly prolonging the residence time of the material at the lesion site [111]. Through the construction of a “stealth” biological interface, such coatings can regulate the immunogenicity of nanomaterials and improve their in vivo persistence, indicating their potential value in serving as long-acting drugs.

These observations indicate that biological challenges remain unavoidable during the clinical translation of nanomaterials, particularly in relation to persistent toxicity and immune incompatibility. In addition, many preclinical studies still rely on simplified in vitro systems or short-term animal experiments, which may not fully predict long-term biodistribution, degradation behavior, chronic inflammatory responses, or patient-to-patient variability in the clinical setting. Fortunately, there are various strategies to reduce the toxicity of nanomaterials, such as biomimetic interface modification and degradable material design. As these approaches become more refined, they are expected to support safer and more effective translation of nanomaterials.

### 5.2. Technical Limitations

Technical limitations are also major obstacles to the clinical translation. Although the methods for fabricating nanomaterials have become increasingly diverse and precise, many of them still lag behind and hinder their practical and scalable application. The lack of large-scale manufacturing technologies, poor clinical compatibility, and insufficient standardization are all urgent technical constraints waiting to be addressed.

#### 5.2.1. Scalability-Precision Trade-Off

Electrospinning is currently one of the most mature techniques for the preparation of nanofibrous materials and has been widely applied in tissue engineering [112,113]. This technique offers several practical advantages, including simple equipment, low cost, and mild reaction conditions. Nanofibers produced by electrospinning typically exhibit small diameters, large specific surface areas, and tunable morphologies, which have made this technique a major focus in fields such as tissue engineering and drug delivery [114].

Despite these advantages, electrospinning still faces important technical limitations. Although it enables the fabrication of nanofibers with controllable structural and processing parameters, the materials often suffer from limited mechanical strength, low production efficiency, and prolonged preparation cycles. This method also cannot simultaneously achieve precise structure control and large-scale preparation. In other words, the technique faces a dilemma between scalability and precision, which substantially limits its industrial translation [113,115]. For specialized architectures such as highly aligned nanofibers, this challenge becomes even more pronounced, as production yield can be very low under the structural control.

A closely relevant challenge is the difficulty of establishing GMP-compliant manufacturing processes for complex nanomaterial systems. Syntheses in lab-scale investigations are generally optimized with flexible experimental parameters. However, clinical translation requires well-documented and tightly controlled production procedures, qualified raw materials, validated devices, environmental monitoring, and traceable quality assurance. For nanomaterials, even subtle changes in solvent exchange, mixing rate, temperature, pH, or storage conditions may alter particle size distribution, surface chemistry, drug loading capacity, scaffold porosity, degradation behavior, and ultimately biological performance. Therefore, a nanomaterial optimized at bench scale may not remain the same product after scale-up.

#### 5.2.2. Batch-to-Batch Reproducibility and Standardization Gap

Technical limitations are also reflected in the growing standardization gap associated with rapid material innovation. International standards, such as those developed by ISO and ASTM, require expert consensus and extensive review before formal adoption. As a result, the revision cycle for such standards often spans 3–5 years. But the current rapid iteration of cutting-edge materials takes only 1–2 years, resulting in a mismatch between standards and innovation.

Such lagging standards lead to the failure of new materials to be incorporated into the standard system. When newly developed materials cannot be incorporated into an updated system in a timely manner, their characterization, quality evaluation, and safety validation may lack a unified basis. Without recognized standards, materials cannot obtain reliable certification, so the clinical translation and industrialization will be extremely slow. These issues have become a persistent impediment to the translation of nanomaterials, particularly for materials whose composition, structure, or surface properties evolve faster than existing evaluation frameworks.

Batch-to-batch consistency represents another hurdle. For translational products, it is not sufficient to demonstrate performance in a single optimized batch. Instead, repeated manufacturing must produce materials with comparable physicochemical properties and biological outcomes. This requirement is challenging for multifunctional nanomaterials, because small batch variations in particle size, surface charge, ligand density, release kinetics, mechanical strength, or nanostructure may cause substantial differences in protein adsorption, immune recognition, cell uptake, and therapeutic efficacy. Consequently, reproducibility should be regarded as a core translational parameter rather than a secondary manufacturing concern. Establishing critical quality attributes, in-process controls, and release criteria will be essential for reducing variability and improving the reliability of nanomaterial-based products.

#### 5.2.3. Sterilization Compatibility, Storage Stability, and Shelf-Life

In addition to fabrication, clinical products must remain stable and retain functionality after sterilization and during storage. This issue is often underestimated in laboratory research, where freshly prepared materials are usually tested directly. In practice, however, sterilization is indispensable for clinical use, and different sterilization methods may affect nanomaterials in different ways. High-temperature steam sterilization may deform polymeric scaffolds or hydrogels and chemical sterilants may leave residues or affect surface activity. Filtration is also not universally suitable, especially for larger particles or complex systems. Therefore, sterilization compatibility should be considered early in material design.

Storage stability and shelf-life are both tightly linked to sterilization requirements. Nanoparticles may aggregate, sediment, undergo surface ligand desorption, or show premature drug release during storage. Likewise, nanofibrous scaffolds and hydrogels may experience deformation, crosslinking changes, or loss of bioactivity over time. If a material requires stringent storage conditions or short-term use after preparation, its clinical applicability may be greatly reduced even when its biological performance is favorable.

### 5.3. Regulatory Pathways

Regulatory pathways are also an indispensable part in ensuring the successful clinical application of nanomaterials. Unified and standardized regulatory criteria can not only reduce detours in material research and development, but also improve product quality and reduce translation risk, thereby facilitating the practical implementation of nanomaterials related biomedical applications.

#### 5.3.1. Critical Quality Attributes

To better regulate the characteristics of the nanomaterials and ensure their safety and effectiveness in medical use, the U.S. Food and Drug Administration (FDA) has emphasized the “Critical Quality Attributes” (CQAs) framework for nanomedicine. CQAs are defined in relation to the intended function of the nanomaterial and its potential influence on product performance. CQAs mainly consist of chemical composition, average particle size, particle size distribution (PSD), general shape and morphology, and stability both physical and chemical.

Meanwhile, any factor that potentially impacts the quality, safety and efficacy of nanoproducts should be considered within the CQAs to support the premarket application. A clear understanding of the CQAs of nanomaterials is therefore essential during product development. Developers and manufacturers of products containing nanomaterial must fully describe the CQAs to ensure their safety and facilitate their clinical applications.

#### 5.3.2. Approved Product Lessons

Approved products can also provide valuable practical guidance for the clinical translation of nanomaterials. One representative example is the use of nanostructured hydroxyapatite (HA) coatings in Stryker’s Tritanium^®^ cages. These scaffolds with Stryker’s proprietary hydroxyapatite (HA) coatings can be used as spinal implants to enhance bone growth. And Stryker’s products have obtained FDA 510(k) clearance and received their market authorization letter, allowing the company to commercialize them under its own name.

Available approval documents indicate that such products have undergone extensive performance evaluation and toxicity testing, and the scopes of application have also been strictly restricted. Stryker’s experience offers great reference for the registration and clinical translation of nanocoated implants. Technological design, experimental testing and clinical value can facilitate the approval of nanomaterials and thus accelerate their clinical translation. More broadly, currently successful examples often involve relatively clear compositions, localized modes of action, and manufacturing processes compatible with established quality control.

### 5.4. Economic Feasibility and Commercialization Consideration

In addition to biological, technical, and regulatory barriers, economic feasibility is another important factor. Many advanced nanomaterials contain multiple functional components and require sophisticated synthesis, purification, sterilization, and characterization procedures. Although these designs may improve biological performance, they may also increase production cost and quality control burden. As a result, a system which is attractive may still face barriers to commercialization if it cannot be manufactured at a reasonable cost.

This issue matters especially in tissue engineering, where products are often designed for large defects, repeated clinical use, or integration into existing surgical routines. If products require expensive raw materials, specialized equipment, and complex transport conditions, their adoption in clinical settings may be limited. Meanwhile, market acceptance depends on whether the product can demonstrate distinctive strengths compared with existing therapies in terms of efficacy, safety, ease of use, and overall cost-effectiveness. Therefore, translational strategies should balance multifunctional material design with economic feasibility and commercialization considerations.

In general, the major reason why many nanomaterial systems do not progress beyond preclinical studies is not the lack of biological activity, but the mismatch between laboratory-level performance and translational requirements. A material may show promising antibacterial, osteogenic, angiogenic, or electroactive functions in vitro and in animal models, yet still fail because of unclear long-term safety, insufficient reproducibility, and poor scalability.

## 6. Future Perspectives

The advances discussed above have highlighted both the remarkable versatility of functional nanomaterials and their growing potential for clinical translation in tissue engineering. Their unique physicochemical and biological properties have enabled a wide range of applications, from structural support and controlled drug delivery to immunomodulation and tissue regeneration. These very properties have also introduced substantial challenges related to manufacturing, biosafety, standardization, and regulation. Even so, functional nanomaterials have already emerged as transformative platforms in tissue engineering, and continued innovation is expected to generate further breakthroughs in the years ahead.

### 6.1. Convergent Technologies

One of the most promising future directions is the convergence of nanotechnology with other frontier biotechnologies, particularly CRISPR-based genome engineering. The clustered regularly interspaced short palindromic repeat (CRISPR) systems have emerged as powerful tools for precise genome editing in diverse biological applications. But CRISPR delivery systems like viral vectors confront the immunogenic responses and toxicity [116]. In contrast with viral vectors, nanosystems with better biocompatibility and lower immunogenicity can deliver the CRISPR systems to the target sites [117]. Thus, the combination of nanomaterials and CRISPR has great genome editing ability in tissue engineering.

For example, by building a bioactive interface, the layer-by-layer (LbL) self-assembling peptide-coated nanofibers provide an efficient platform for the delivery of CRISPR/dCas9 systems for neural tissue engineering [118]. Such strategies suggest that future nanomaterials may evolve beyond passive carriers toward more adaptive and biologically interactive systems.

### 6.2. Sustainable Development

Alongside functional performance, sustainability is likely to become an increasingly important criterion in the future development of nanomaterials. As the world faces enormous challenges in global climate and energy, the field should not only focus on the application of nanomaterials but also consider the sustainability of their development. Currently, the use of chemicals and high energy requirements for synthesis stand as an environmental hazard.

Fortunately, green synthesis offers an eco-friendly and sustainable route for producing nanomaterials. Tariq et al. demonstrated that plant extracts such as Sonchus oleraceus can serve as effective reducing and stabilizing agents for the fabrication of ZnO@GO nanocomposites, eliminating the need for toxic chemicals. This approach can yield nanomaterials with favorable surface properties and significant antibacterial performance, highlighting the potential of green chemistry in advanced material design [119]. Beyond plant extracts, naturally derived proteins such as ovalbumin from egg white have emerged as effective biotemplates for the green synthesis of metal oxide nanostructures. Studies have demonstrated that ovalbumin mediates the formation of ZnO and MgO nanoparticles with tunable crystallite sizes (ranging from 7.9 to 13.5 nm) and antibacterial activity, enabling sustainable nanomaterial production [120]. Serving as a viable alternative to conventional chemical and physical fabrication strategies, these green approaches feature low toxicity and improved environmental sustainability.

Despite exploring greener methods, sustainability metrics should be established. Life Cycle Assessment (LCA) is a key method which enables a comprehensive evaluation of the entire process from production to application and disposal. Based on LCA, green synthesis and degradable design can be optimized, thereby reducing the consumption and environmental risks associated with nanomaterial production. In the future, broader implementation of LCA across the full life cycle of nanomaterials may provide important support for the sustainable advancement of tissue engineering.

### 6.3. Global Translation Networks

Another significant future direction involves the establishment of broader international collaboration and multi-center validation networks for nanomaterial translation. As the field continues to expand rapidly, countries have attached increasing importance to nanotechnology research, industrialization, and biomedical application. In recent years, China has hosted many global academic events on nanomaterials, such as the Directors’ Forum of the Global Nanoscience and ChinaNANO2025. These events have not only provided global exchange platforms for scientists; they also emphasize the emerging opportunities for nanotechnology in the AI era, such as the application of AI in nanomedicine design.

At the same time, the transformation of nanomaterials has been continuously improved. According to the China Nanotechnology Industry White Paper (2025), from 2000 to 2025, the total number of granted nanotechnology patents worldwide exceeded 1.078 million, of which China accounted for 464,000, representing as high as 43% and ranking first in the world. These data have reflected the rapid expansion of innovation capacity and suggested that large-scale validation, industrial translation, and regulatory alignment may become increasingly feasible.

Looking ahead, global clinical trial networks and multi-center translational platforms may play a crucial role in accelerating the application of functional nanomaterials. Such frameworks could help improve reproducibility, harmonize evaluation criteria, and strengthen the clinical evidence. In parallel, the incorporation of artificial intelligence, big data, and intelligent manufacturing into nanomaterial design and evaluation may further improve development efficiency and translational success.

Functional nanomaterials are reshaping the landscape of tissue engineering by offering unprecedented opportunities to modulate cellular behavior, reconstruct tissue microenvironments, and enhance regenerative outcomes. Their integration into bioactive interfaces, scaffolds, and hybrid systems has already demonstrated substantial potential across multiple tissue types (Table 2). Despite the considerable challenges that remain in biosafety, manufacturing, standardization, and clinical translation, advances in interdisciplinary research are steadily expanding the feasibility of their practical application. Looking forward, the successful integration of innovative material design with rigorous translational evaluation will be essential for unlocking the full clinical value of functional nanomaterials and advancing the future of regenerative medicine.

## Figures and Tables

**Table 1:** Comparative evaluation of major nanomaterials used in tissue engineering.

Nanomaterials	Regenerative functions	Advantages	Limitations	Applications	Ref.
Nanoparticles	Drug or growth factor delivery, ROS modulation, antibacterial action, angiogenesis regulation, immunomodulation	Flexible formulation, adaptable to different disease microenvironments, suitable for local or systemic delivery	Dose-dependent toxicity, burst release, uncertain long-term biodistribution, difficult in vivo behavior prediction	Bone, skin, neural, and cardiac applications	[35,36,58,62,81]
Nanofibers and nanofibrous scaffolds	Structural support, cell adhesion, directional guidance, local release support	Strong biomimicry, effective for cell guidance and tissue integration, well suited for localized implantation	Scale-up challenges, reproducibility issues for highly aligned architectures, sterilization constraints	Bone, skin, and neural regeneration	[21,41,71,76]
MXene-based materials	Electrical stimulation, cell signaling modulation, antibacterial support, multifunctional scaffold reinforcement	Particularly effective for electroactive tissues, integration into composite systems, strong multifunctional potential	Oxidation sensitivity, unpredictable degradation behavior, limited long-term biosafety, unclear regulatory pathway	Neural and cardiac regeneration; bone repair	[41,42,43]
Nanocrystalline mineral systems	Mineralization, osteoinduction, implant interface activation, support for growth-factor delivery	Strong relevance to bone biology, clearer mechanism, compatibility with bone substitutes	Limited use beyond hard tissue, uncertain bioactivity, interface stability concerns	Bone regeneration	[53,54,55]
Liposomal and lipid-based systems	Targeted delivery, prolonged retention, immune evasion, biochemical modulation	Potential for drug or gene delivery, relatively mature pharmaceutical framework, adaptable for targeting	Formulation instability, variable encapsulation efficiency, complex quality control for biomimetic coatings	Cardiac and neural delivery; broader potential across tissues	[82]
Conductive carbon-based nanomaterials	Electrical coupling, neurite outgrowth, cardiomyocyte maturation, scaffold strengthening	Useful for electroactive tissues, improve both conductivity and mechanics in composites	Variable purity and surface chemistry, uncertain long-term persistence and clearance	Neural and cardiac regeneration	[32,80]
Multifunctional hydrogels and nanocomposites	Combined structural support, drug release, inflammation control, angiogenesis, conductivity, wound adaptation	Versatile and clinically appealing for tissue repair, especially in soft tissues	Mechanistic complexity, difficult standardization, batch variability, challenging product regulation	Skin, neural, and cardiac regeneration	[33,66,71,80]

**Table 2:** Comparison of conventional materials and nanomaterials in tissue regeneration.

Dimension	Conventional Materials	Nanomaterials	Advantages of Nanomaterials	Current Limitations of Nanomaterials
Structural scale and biomimicry	Usually micro-/macro-scale; limited ability to mimic the native extracellular matrix (ECM) structure	Nanoscale features; resemble natural ECM components and hierarchical tissue structure	Better biomimicry, enhanced cell modulation, and improved microenvironmental regulation	Nanoscale complexity may reduce reproducibility and increase manufacturing difficulty
Cell–material interaction	Primarily passive support; weak control over cell adhesion, migration, and differentiation	Active regulation of cell fate through nanotopography, surface charge, and ligand presentation	More precise modulation of cellular behaviors and better interaction	Cell responses may vary with particle size, morphology, charge, and dose, complicating optimization
Surface bioactivity	Often bioinert or moderately bioactive without further modification	High surface-area-to-volume ratio enables efficient protein adsorption, growth factor loading, and surface functionalization	Improved bioactivity and stronger interface with host tissue	Excessive surface bioactivity may trigger unintended protein corona formation or off-target biological effects
Mechanical adaptability	Fixed or limited mechanical properties; difficult to match dynamic tissue healing stages	Designed with tunable stiffness gradients and shape-memory behavior	Better adaptation to specific mechanical demands and dynamic regeneration processes	Mechanical optimization may require complex design and advanced fabrication
Multifunctionality	Typically provides only structural support or a single function	Integrates multiple functions, such as antibacterial activity, conductivity, antioxidation, sensing, and drug delivery	Enables smart regenerative platforms with synergistic therapeutic effects	Multifunctional integration increases synthesis complexity and regulatory burden
Drug/growth factor delivery	Conventional carriers often show burst release, low targeting efficiency, and poor temporal control	Stimuli-responsive nanocarriers enable sustained, sequential, or on-demand release (e.g., pH-, thermal-, glucose-responsive nanosystems)	Improved delivery of bioactive molecules such as BMP-2 and VEGF	Risk of unstable release kinetics, carrier accumulation, and challenges in dose standardization
Manufacturing precision	Established manufacturing routes but lower structural precision	Advanced methods such as two-photon polymerization and microfluidics enable high-precision nanoengineering	Fine control over architecture, interface, and function	High precision often comes at the expense of throughput, scalability, and cost
Scalability and industrial production	Generally mature, standardized, and easier to produce in large volumes	Nanomanufacturing is often difficult to scale while maintaining consistency	Potential for high-performance products if they can be manufactured at scale	Scalability–precision dilemma; batch variation and low yield
Biocompatibility and long-term safety	More extensively studied and generally better applied clinically	Some nanomaterials show excellent short-term performance but have long-term nano toxicity	Opportunity to engineer safer surfaces, including immune stealth and bioinspired coatings	Risks include persistence, mitochondrial dysfunction, immune reactions, and nanotoxicity
Regulatory readiness	Clearer standards and more established translation pathways	Regulatory frameworks for nanomaterials are still evolving	Early alignment with critical quality attributes may accelerate translation	Standardization gaps and regulatory ambiguity delay clinical application
Cost and accessibility	Lower cost and more accessible for routine clinical use	Higher development and production costs due to sophisticated design and characterization	Offer superior therapeutic benefit in complex or refractory cases	Economic burden may limit widespread adoption before commercialization matures
Clinical translation status	Many conventional biomaterials already have substantial clinical history	Some nanoproducts show promise, but most remain preclinical or in early stage of translation	Strong potential for next-generation regenerative medicine and personalized therapy	Requires more multi-center validation, long-term safety data, and standardized quality control

## Data Availability

No new data were created or analyzed in this study. Data sharing is not applicable to this article.
